# Design, synthesis, and anti-breast cancer activity evaluation of novel 3-cyanopyridine derivatives as PIM-1 inhibitors

**DOI:** 10.1007/s11030-024-11010-8

**Published:** 2024-11-09

**Authors:** Bahgat R. M. Hussein, Hayam H. Mohammed, Eman A. Ahmed, Omar Alshazly, Mamdouh F. A. Mohamed, Omran A. Omran

**Affiliations:** 1 Department of Chemistry, Faculty of Science, Sohag University, Sohag, 82524 Egypt; 2 Department of Pharmaceutical Chemistry, Faculty of Pharmacy, Sohag University, Sohag, 82524 Egypt

**Keywords:** Cyanopyridines, Regioselective, Anticancer, PIM inhibitors, Molecular docking studies, Breast cancer

## Abstract

A novel series of cyanopyridines **7a-j** were synthesized via a one-pot multicomponent reaction of arylidene **4** with ammonium acetate **5** and respective methylaryl/heterylketones **6a-j** in ethanol using vanillin as a natural starting material. Moreover, the regioselective alkylation reaction was studied by the treatment of cyanopyridines **7a-f** and **7j** with CH_3_I in the presence of K_2_CO_3_ in DMF to afford *O*-methylcyanopyridines **8a-g** (major) and *N*-methylcyanopyridines **9a-g** (minor), whereas bipyridine **7h** gave bipyridinium iodide salt **10**. All of the designed cyanopyridines were evaluated as anti-breast cancer (MCF-7) cell lines via PIM Kinase inhibitory activity, and the results displayed that some of them showed high activities, especially compounds **7h** and **8f**, which showed excellent activities against MCF-7 with IC_50_ values of 1.89 and 1.69 μM, respectively, more potent than the reference drug doxorubicin. Mechanistically, compounds **7h** and **8f** exhibited strong in vitro PIM-1 kinase inhibitory activity with an IC_50_ of 0.281 and 0.58 μM, respectively, compared to the reference staurosporine. Moreover, compound **7h** arrested the tumor cells at the S phase and caused cell death mainly by inducing early and late apoptosis. Molecular docking studies against PIM-1 revealed good binding modes of the synthesized compound and showed agreement with the biological results.

**Graphical abstract:**

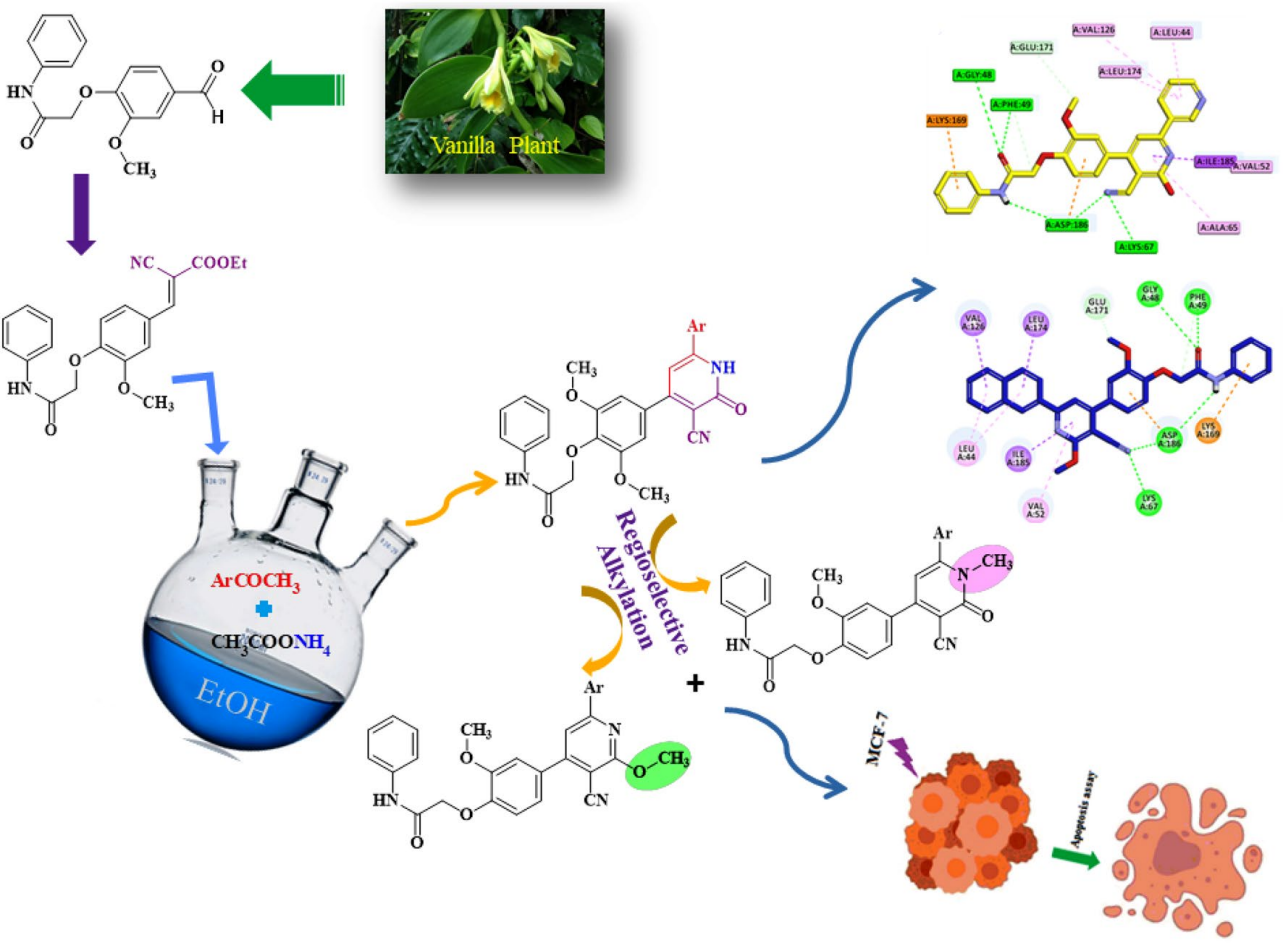

## Introduction

With an estimated 15 million deaths per year by 2030, cancer poses a significant crisis to global public health and healthcare systems. Initially regarded as a genetic disease, it is now widely recognized that cancer is a complex condition influenced by both genetic and epigenetic factors [1]. The intricate signaling networks involved in cancer necessitate the perturbation of multiple targets simultaneously, as cancer cells can employ diverse compensatory pathways for their survival [2, 3].

PIM-1, a serine/threonine kinase belonging to the PIM (proviral insertion site in Moloney murine leukemia virus) kinase family, is considered a proto-oncogene. This family includes two other isoforms, PIM-2 and PIM-3 [4, 5]. PIM-1 exerts a critical role in cell signaling pathways and various cellular functions, including cell cycle regulation, cell survival, proliferation, apoptosis, and drug resistance. It achieves this by phosphorylating and regulating the activity of numerous proteins involved in these processes [6–9]. PIM-1 has demonstrated higher expression levels in various solid cancers, including prostate, colon, hepatic, pancreatic, and breast cancers, as well as hematological cancers like leukemia, multiple myeloma, and diffuse large B cell lymphomas (DLBCL) [10–16]. It has also been observed in circulating tumor cells (CTCs) from patients with metastatic castration-resistant prostate cancer (mCRPC) [17]. Notably, its expression is absent in benign tumors. Overexpression of PIM-1 has been linked to cancer initiation and progression through three significant mechanisms: inhibiting apoptosis, promoting cell proliferation [18, 19], and promoting genomic instability [20]. In vitro studies have shown that PIM-1 overexpression enhances tumor growth and confers resistance to drug-induced apoptosis in cancer cells [21]. The degree of PIM-1 overexpression correlates with tumor grade and neoplastic transformation [22]. Furthermore, inhibiting these isoforms of kinases in mouse experimental models did not exhibit significant side effects [23]. Moreover, specific, and potent inhibitors of PIM-1 kinase have been identified, which have been shown to induce apoptotic cell death, sensitize cancer cells to chemotherapy, and synergize with other anti-tumor agents.

**Fig. 1:**
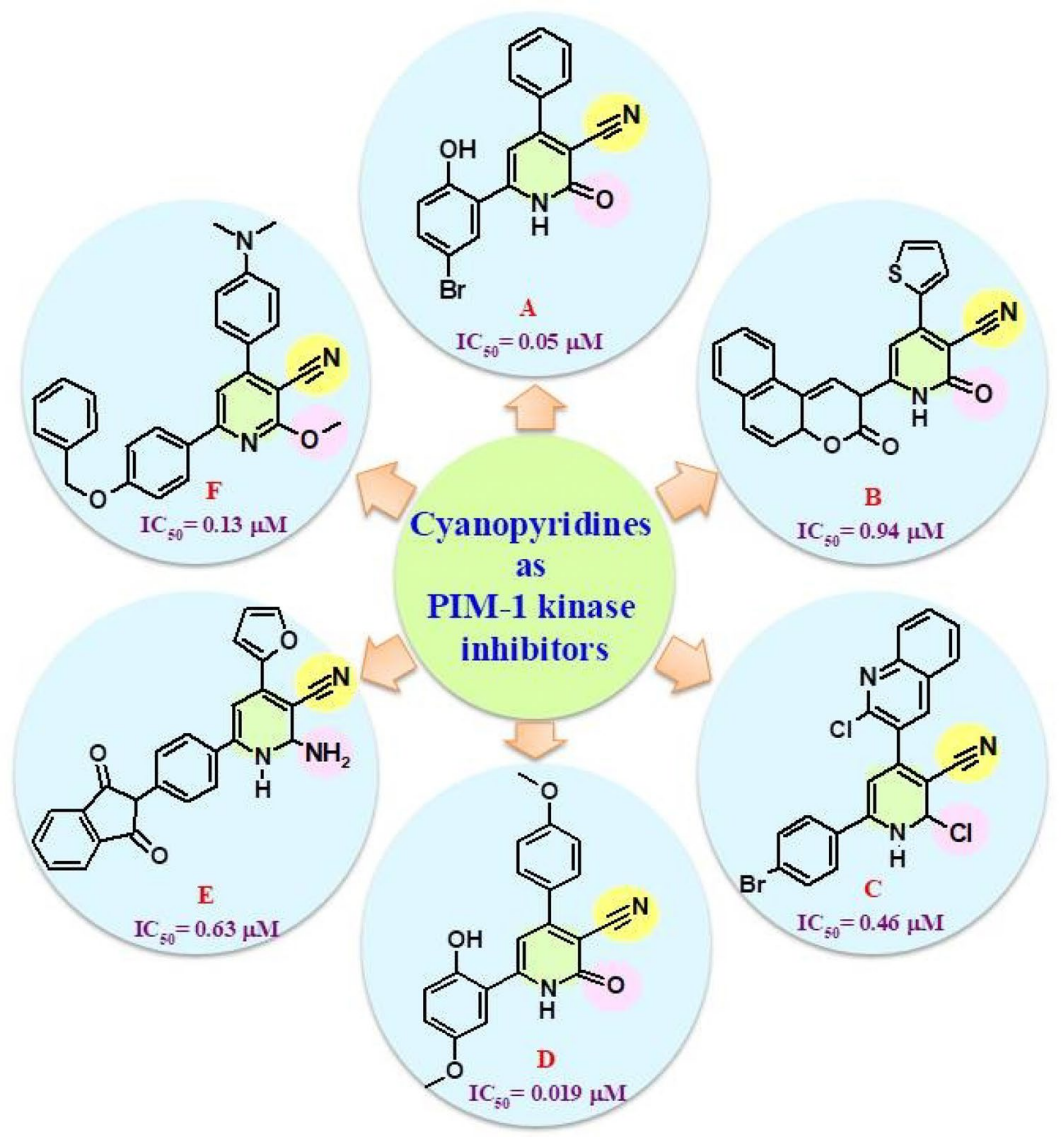
Some of the cyanopyridines have high PIM-1 kinase inhibitory activity

On the other hand, many of the cyanopyridnes were reported as promising anticancer agents with high PIM-1 kinase inhibitory activity, such as compound **A** (IC_50_ = 0.05 μM) [24], compound **B** (IC_50_ = 0.94 μM) [25], compound **C** (IC_50_ = 0.46 μM) [26], compound **D** (IC_50_ = 0.019 μM) [27], compound **E** (IC_50_ = 0.63 μM) [28], and compound **F** (IC_50_ = 0.13 μM) [29] (Fig. 1). Therefore, PIM-1 kinase represents an attractive therapeutic target [20, 30], and inhibiting it presents an intriguing strategy for treating various tumor types by inducing apoptosis and suppressing proliferation [22].

Thus, from the above mention information and in continuation of our previous work [31–36], we designed and synthesized a novel series of cyanopyridines **7a-j** via one-pot multicomponent reaction, *O*-methylcyanopyridines **8a-g**, and *N*-methylcyanopyridines **9a-g** via alkylation reaction. The in vitro cytotoxicity of the target compounds were screened against the breast cancer (MCF-7) cell line using the MTT assay method. Furthermore, the PIM-1 kinase inhibitory activity for the most potent cyanopyridines (**7h** and **8f**) was investigated. The most potent compound **7h** was investigated for apoptosis and cell cycle progression. The molecular docking was studied against PIM-1 (PDB ID: 2OH4). Finally, the study of the structure–activity relationship (SAR) was determined.

## Results and discussion

### Chemistry

**Scheme 1:**
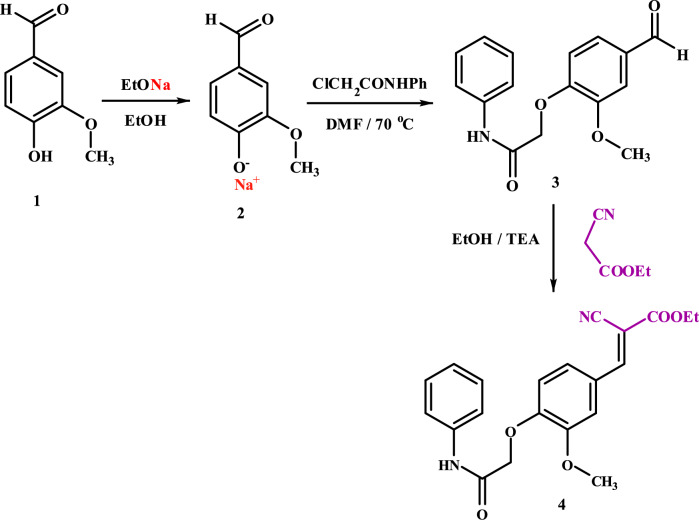
Synthesis of* O*-alkyl vanillin **3** and arylidene **4**

Herein, we used a simple method for the synthesis of *O*-alkyl vanillin **3** [37, 38] from vanillin **1** (an available and inexpensive natural product) by stirring it with ethanolic NaOEt to give vanillin sodium salt **2**, which was isolated and subjected to react with 2-chloro-*N*-phenylacetamide as an active halo-compound in dry dimethylformamide at 70 ^ο^C. The synthetic *O*-alkyl vanillin **3** undergoes Knoevenagel condensation reaction when it is reacted with ethyl cyanoacetate in the presence of TEA as a catalyst to afford the arylidene **4** in ethanol (Scheme 1).

The chemical structures of the newly synthesized compounds were proven by the spectral (IR, ^1^H NMR) and elemental analyses. The IR spectrum of vanillin sodium salt **2** showed absorption bands corresponding to CH aromatic at 3052 cm^−1^, CH aliphatic at 2971, 2936 cm^−1^, and C = O of the formyl group at 1669 cm^−1^. Its ^1^HNMR spectrum showed the disappearance of the OH group and the appearance of two singlet signals corresponding to CHO and OCH_3_ at 9.31 and 3.71 ppm, as well as one singlet signal at 7.02 ppm and two doublet signals at 7.15 and 6.31 ppm due to aromatic protons with a coupling constant of *J* = 7.9 Hz. The ^13^C NMR of vanillin sodium salt **2** showed two signals at 187.1 and 55.1 ppm corresponding to C = O_formyl_ and OCH_3_, respectively. As well as the aromatic *sp*^*2*^ carbons are characterized by signals at 170.6, 151.7, 130.9, 118.7, 117.8, and 108.0 ppm.

**Scheme 2:**
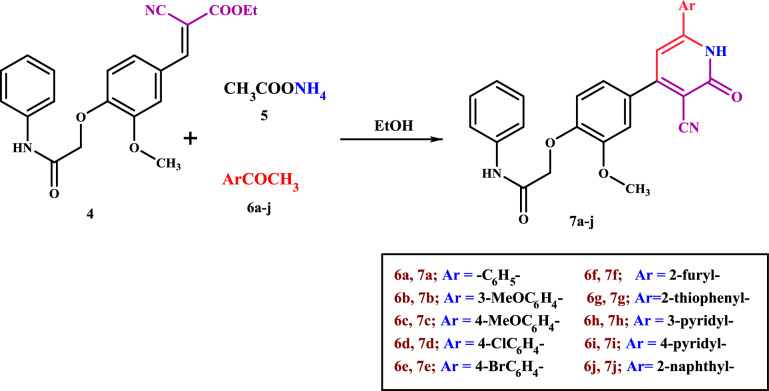
The synthesis of cyanopyridines **7a-j** via one-pot multicomponent reaction

Moreover, a novel series of cyanopyridines **7a-j** were synthesized via one-pot multicomponent reaction (MCR) of arylidene **4** with ammonium acetate **5** and respective methylaryl/heterylketones **6a-j**, namely acetophenone, 3-methoxyacetophenone, 4-methoxyacetophenone, 4-chloroacetophenone, 4-bromoacetophenone, 2-acetylfuran, 2-acetylthiophene, 3-acetylpyridine, 4-acetylpyridine, and/or 2-acetylnaphthalene, respectively, in ethanol (Scheme 2).

The structures of cyanopyridines **7a-j** were confirmed by spectral analyses, which showed the absence of CH_olefinic_ group with the appearance of a new NH group and aryl group. For example, the IR spectrum of cyanopyridine **7a** showed absorption bands corresponding to NH at 3390 cm^−1^, CH aromatic at 3076 cm^−1^, CH aliphatic at 2969 and 2917 cm^−1^, C≡N at 2217 cm^−1^, and C = O amide at 1690 cm^−1^. Its 1H NMR spectrum showed four singlet signals corresponding to 2NH, CH_2_, and OCH_3_ at 12.62, 10.02, 4.81, and 3.92 ppm, beside CH of aromatic protons as two singlet signals at 7.42, 6.86 ppm, respectively; three doublets at 7.90, 7.63, and 7.15 with a coupling constant of 6.8, 7.8, and 8.4 Hz, respectively; two triplet signals 7.34, and 7.09 with a coupling constant of 7.3 Hz, and one multiplet 7.57–7.53 ppm. Its ^13^C NMR revealed the appearance of four signals of two C = O_amide_, CH_2_, and OCH_3_ at 166.7, 162.6, 68.6, and 56.4 ppm, respectively; and the rest of aromatic *sp*^*2*^ carbons appeared at 159.8, 151.7, 149.8, 149.4, 138.7, 132.9, 131.5, 129.8, 129.3, 129.2, 128.1, 124.2, 121.8, 120.0, 117.2, 114.4, 113.1, 106.6, and 90.3 ppm.

**Scheme 3:**
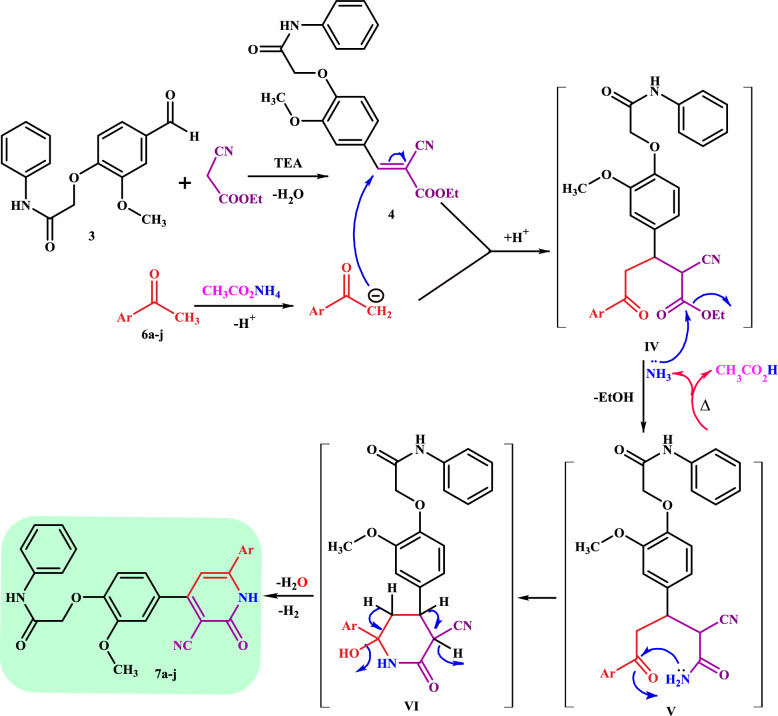
The suggestion reaction mechanism for the formation of the cyanopyridines **7a-j**

The plausible reaction mechanism for the synthesis of pyridines **7a-j** can be explained by the formation of the arylidene **4** via the Knoevenagel condensation reaction of *O*-alkyl vanillin **3** with ethyl cyanoacetate in basic medium. The arylidene **4** undergoes a nucleophilic addition reaction by attacking the enolate ion of the activated methylaryl/heterylketone **6a-j** in the presence of ammonium acetate to afford the intermediate **IV**, followed by nucleophilic addition of ammonia (produced from the decomposition of CH_3_CO_2_NH_4_) on the C = O_ester_ group to form the intermediate **V** with the liberation of the EtOH molecule. The intermediate **V** undergoes the intramolecular cyclization reaction of the amino group to another C = O group via a Michael addition reaction to give intermediate **VI**, which is easily aromatized by the dehydration and deprotonation to afford the target products (Scheme 3).

**Scheme 4:**
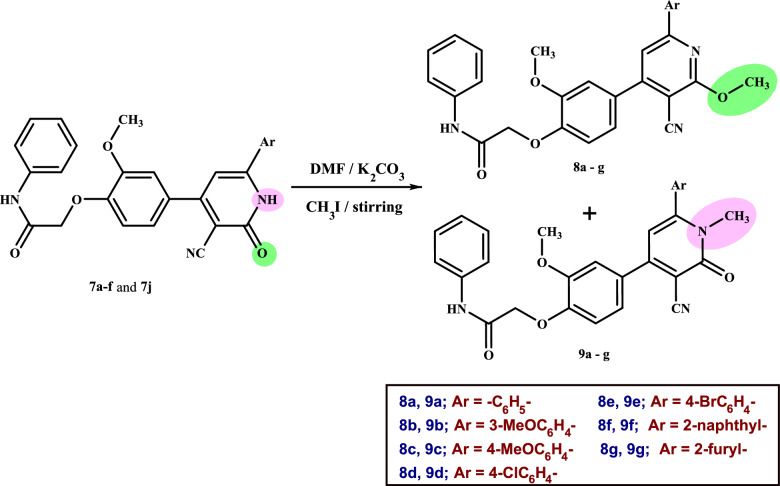
Synthesis of *O*-methylcyanopyridines **8a-g** and *N*-methylcyanopyridines **9a-g**

Furthermore, the treatment of cyanopyridines **7a-f** and **7j** with methyl iodide in the presence of potassium carbonate as a catalyst undergoes regioselective alkylation reactions in dry DMF to afford two new series of *O*-methylcyanopyridines **8a-g** and *N*-methylcyanopyridines **9a-g** (Scheme 4). The products were separated by thin layer chromatography using a mixture of chloroform and petroleum ether (9:1) as a suitable eluent; the* O*-methylcyanopyridines were separated as a major product (Rf = 0.10:0.20), whereas the *N*-methylcyanopyridines were separated as a minor product (Rf = 0.23:0.47) (**see Experimental part**).

The IR spectrum of *O*-methylcyanopyridine **8b** (as an example) showed the appearance of an absorption band corresponding to NH_,_ CH aromatic, CH aliphatic, C≡N and C = O amide at 3396, 3067, 2944–2843, 2218, and 1693 cm^−1^, respectively. Its ^1^H NMR spectrum showed the disappearance of the NH signal and the appearance of five singlet signals at 10.13, 4.83, 4.15, 3.93, and 3.86 ppm due to NH, CH_2_, and three OCH_3_, respectively. In addition, Ar–H protons split into two singlets at 7.44 and 7.34 ppm, one doublet with a coupling constant of 4.2 Hz at 7.65 ppm, and two multiplets at 7.82–7.78 and 7.16–7.10 ppm, respectively. Also, its ^13^C NMR spectrum showed C = O_amide_, CN, CH_2_, and three OCH_3_ signals at 166.7, 116.1, 68.4, 56.3, 55.7, and 54.9 ppm, respectively, and the residual signals appeared at 164.7, 160.1, 157.3, 156.4, 149.5, 149.0, 149.4, 138.8, 138.6, 130.4, 129.4, 129.2, 124.1, 121.9, 120.3, 119.9, 116.5, 114.2, 114.1, 113.3, and 92.5 ppm assigned to *sp*^*2*^ aromatic carbons.

Also, the *N*-methylcyanopyridine of **9b** (as another example) showed in the IR spectrum absorption bands corresponding to NH at 3392 cm^−1^, CH aromatic at 3072 cm^−1^, CH aliphatic at 2960, 2940, and 2839, C≡N at 2219 cm^−1^, and C = O amide at 1684 cm^−1^. Its ^1^H NMR spectrum revealed the disappearance of one of NH and the appearance of five singlet signals corresponding to NH, CH_2_, two OCH_3_, and NCH_3_ at 10.14, 4.82, 3.89, 3.82, and 3.35 ppm, respectively. Beside the aromatic protons appeared in the range 7.84–6.53 ppm. As well, its ^13^C NMR revealed the appearance of six signals of C = O_amide_, CN, CH_2_, two OCH_3_, and NCH_3_ at 166.6, 116.2, 68.3, 56.2, 55.8, and 35.1 ppm, respectively; and the residual of aromatic *sp*^*2*^ carbons appeared at 161.5, 159.7, 157.6, 154.4, 149.9, 149.3, 138.8, 135.8, 130.4, 129.2, 128.9, 124.1, 121.8, 120.9, 119.8, 117.4, 114.4, 113.9, 112.7, 108.8, and 98.1 ppm. The Dept-135 NMR spectrum of **9b** confirmed his structure because it showed the appearance of CH_2_ in a negative direction at 68.6 ppm and the fourteen Ar–H carbons and two OCH_3_ and NCH_3_ appeared in a positive direction at 130.4, 129.2, 124.1, 121.8, 120.9, 119.8, 116.2, 114.4, 113.8, 112.7, 108.8, 56.2, 55.8, and 35.1 ppm, respectively.

On the other hand, the alkylation of bipyridine **7 h**, which contains two pyridine rings in the same conditions, undergoes two folds of CH_3_I on the OH of the cyanopyridine ring and nitrogen on another one, giving only one product, bipyridinium iodide salt **10 (Eq. **1)*.*1

Synthesis of pyridinium iodide salt 10.

The IR spectrum of bipyridinium iodide salt **10** showed the absorption bands corresponding to the NH group at 3178 cm^−1^, CH aromatic group at 3061 cm^−1^, CH aliphatic group at 2952, and 2830 cm^−1^, Cyano group at 2220 cm^−1^ and C = O amide group at 1689 cm^−1^. Its ^1^H NMR spectrum revealed the appearance of five singlet signals at 10.21, 4.86, 4.48, 4.23, and 3.92 ppm due to NH, CH_2_, N^+^CH_3_, and two OCH_3_, respectively. The aromatic protons were divided into three singlet signals at 9.81, 8.15, and 7.48 ppm, three doublet signals at 9.37, 9.11, and 7.64 ppm with a coupling constant of 8.1, 5.8, and 7.8 Hz, respectively, one triplet at 8.31 ppm with a coupling constant of 7.0 Hz, and two multiplets at 7.40–7.32 and 7.19–7.08 ppm. The ^13^C NMR spectrum of bipyridinium iodide salt **10** appeared six signals at 166.6, 115.8, 68.5, 56.6, 55.6, and 48.9 for C = O_amidic_, CN, CH_2_, two OCH_3_, and N^+^CH_3_, respectively. The residual signals appeared at 165.1, 157.2, 150.9, 150.0, 149.6, 146.6, 145.1, 143.1, 138.8, 136.5, 129.1, 128.8, 128.2, 124.1, 122.2, 119.9, 115.5, 114.5, 113.5, and 95.2 ppm due to aromatic *sp*^*2*^ carbons.

**Fig. 2:**
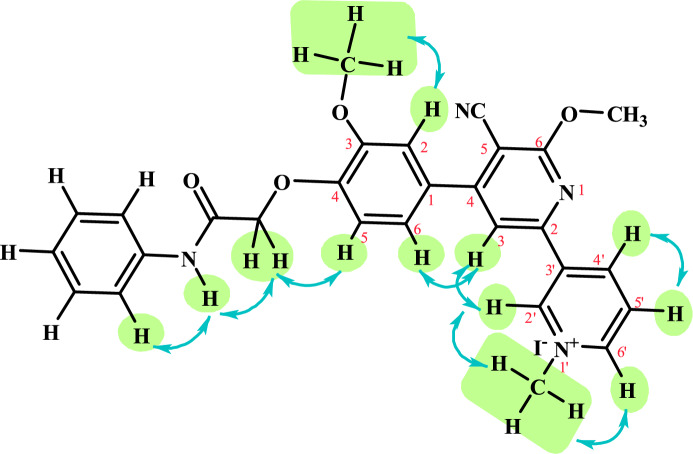
The correlation peaks between the protons by the NOESY NMR spectrum of compound **10**

The structure of compound **10** was confirmed by the NOESY NMR spectrum. The pattern of NOE contacts showed the correlation peaks between the protons of compound **10** which observed strong interaction between OCH_3_ with H-2, N^+^CH_3_ with H-2' (Py) & H-6' (Py), CH_2_ with NH & H-5, H-3 (Cy-Py) with H-6 & H-2' (Py), NH with H-Ph and H-4' (Py) with H-5' (Py) (see **SI**, Fig. 2).

### Biology

#### Anticancer activity

##### Anti-proliferative activity:

**Fig. 3:**
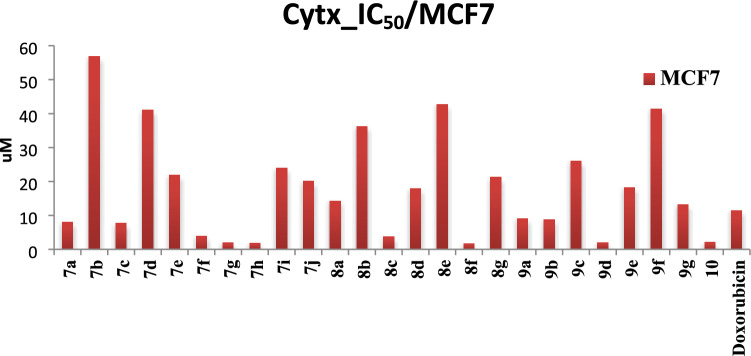
The IC_50_ (µM) of antiproliferative assays of Doxorubicin and the target compounds against MCF-7 human cancer cell lines

In the present study, all synthesized cyanopyridine hybrids were subjected to in vitro cytotoxicity testing using the MTT assay method against MCF-7 human cancer. Notably, our findings revealed the existence of distinct groups with differing levels of activity when compared to the reference drug, doxorubicin. Firstly, a group of compounds displaying remarkably potent activity that far surpassed the efficacy of doxorubicin’s IC_50_ value of 11.49 ± 0.47 µM, the established reference drug. This group exhibited a superior cytotoxic effect, including 3-cyano-2-methoxypyridine derivatives, particularly compounds **8f** and **10** containing naphthyl and 3-pyridyl moieties, respectively, which demonstrated the highest cytotoxic activity with IC_50_ values of 1.69 ± 0.07 µM and 2.13 ± 0.09 µM, respectively. The cyano-2-methoxypyridine derivative with 4-methoxyphenyl group **8c** displayed significant antiproliferative activity with IC_50_ = 3.74 ± 0.15 µM but less than the naphthyl and 3-pyridyl derivatives. Moreover, 3-cyanopyrid-2-one derivatives bearing 3-pyridyl **7h**, furan **7f**, and thiophenyl **7g** groups displayed significant cytotoxicity, with IC_50_ values of 1.89 ± 0.08 µM, 3.98 ± 16 µM and 1.92 ± 0.08 µM, respectively. Additionally, the 3-cyano-1-methylpyrid-2-one derivative **9d**, containing a 4-chlorophenyl group, exhibited notable cytotoxicity, with an IC_50_ value of 2.05 ± 0.08 µM (Table 1**, **Fig. 3).

**Table 1:** The IC_50_ (µM) of antiproliferative assays of the target cyanopyridine derivatives **7a-j**, **8a-g**, **9a-g**, and **10**

Ser	Sample				Cytotoxicity IC_50_/uM, SD ( ±)
	Code	Structure	Ar	M.W (g/mol)	MCF7
1	7a		C_6_H_5_-	451.47	8.0617 ± 0.33
2	7b		3-MeO-C_6_H_4_-	481.49	56.813 ± 2.34
3	7c		4-MeO-C_6_H_4_-	481.49	7.7251 ± 0.32
4	7d		4-Cl-C_6_H_4_-	485.91	41.126 ± 1.69
5	7e		4-Br-C_6_H_4_-	530.36	21.907 ± 0.9
6	7f		2-Furyl	441.43	3.9819 ± 0.16
7	7 g		2-Thiophenyl	457.50	1.928 ± 0.08
8	7 h		3-pyridyl	452.46	1.8937 ± 0.08
9	7i		4-pyridyl	452.46	23.95 ± 0.98
10	7j		2-Naphthyl	501.53	20.094 ± 0.83
11	8a		C_6_H_5_-	465.49	14.28 ± 0.59
12	8b		3-MeO-C_6_H_4_-	495.52	36.192 ± 1.49
13	8c		4-MeO-C_6_H_4_-	495.52	3.7466 ± 0.15
14	8d		4-Cl-C_6_H_4_-	499.94	17.973 ± 0.74
15	8e		4-Br-C_6_H_4_-	544.39	42.638 ± 1.75
16	8f		2-Naphthyl	515.55	1.6966 ± 0.07
17	8 g		2-Furyl	455.46	21.251 ± 0.87
18	9a		C_6_H_5_-	465.49	9.1005 ± 0.37
19	9b		3-MeO-C_6_H_4_-	495.52	8.7957 ± 0.36
20	9c		4-MeO-C_6_H_4_-	495.52	26.07 ± 1.07
21	9d		4-Cl-C_6_H_4_-	499.94	2.0534 ± 0.08
22	9e		4-Br-C_6_H_4_-	544.39	18.285 ± 0.75
23	9f		2-Naphthyl	515.55	41.421 ± 1.7
24	9 g		2-Furyl	455.46	13.199 ± 0.54
25	10		–	608.42	2.1387 ± 0.09
***	Doxorubicin	–	–	543.52	11.495 ± 0.47

Secondly, another group of compounds demonstrated comparable activity to that of doxorubicin. This finding suggests that these compounds possess a similar level of cytotoxic potential. Including 3-cyanopyrid-2-one derivatives **7a** and 7**c** bearing phenyl and 4-methoxy phenyl respectively and showing IC_50_ = 8.06 ± 0.33, 7.72 ± 1.69 µM respectively. The 3-cyano-1-methylpyrid-2-one derivatives **9a** and **9b** containing phenyl and 3-methoxyphenyl respectively, showed similar cytotoxicity with IC_50_ of 9.10 ± 0.37 and 8.79 ± 0.36 µM. The remaining derivatives displayed varying degrees of cytotoxic activity, their IC_50_ values ranged from 13.9 to 56.8 µM, indicating moderate to weak efficacy in inhibiting cell growth.

From the results, it is obvious that 3-cyanopyridin-2-one derivatives showed similar activities as the derivatives that alkylated with methyl group on both nitrogen and oxygen groups. It was observed that the aromatic substituent on the cyanopyridine ring exerted varied cytotoxic activity.

**Fig. 4:**
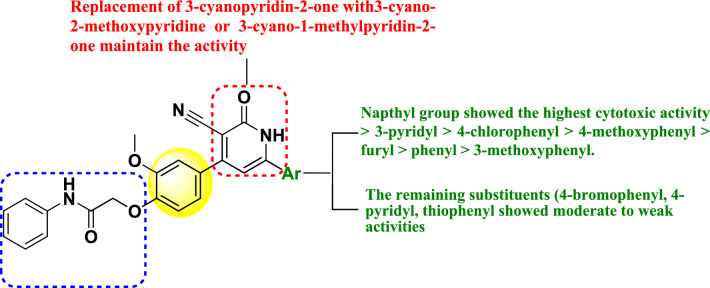
SAR of the cytotoxicity of the synthetic cyanopyridine derivatives

Among the various substituents considered, the naphthyl group demonstrated the highest level of cytotoxic activity, followed by the 3-pyridyl group. Subsequently, the 4-chlorophenyl substituent exhibited a lower degree of cytotoxicity, succeeded by the 4-methoxyphenyl, followed by furan and phenyl substituents, followed by the 3-methoxyphenyl substituent, while the remaining substituent showed a varied range of weak to moderate activities. These findings can be succinctly represented in Fig. 4, illustrating the cytotoxicity structure activity relationship (SAR) of the cyanopyridine derivatives.

##### PIM1 Kinase inhibition activity:

**Fig. 5:**
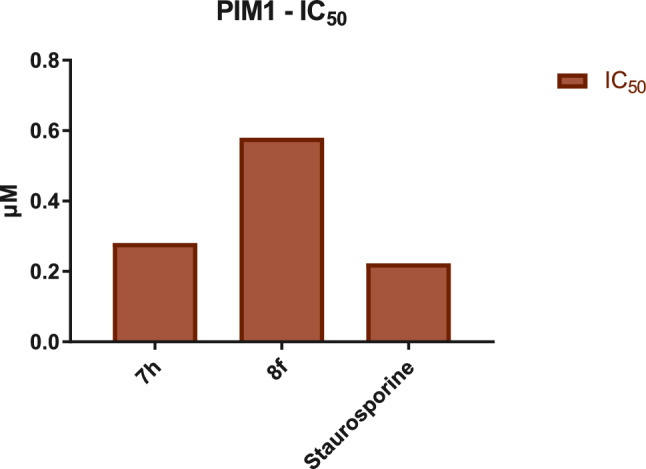
The IC_50_ (µM) of antiproliferative assays of Staurosporine and the target compounds against PIM1 Kinase enzyme

In accordance with the findings from the in vitro cytotoxicity study, the evaluation of PIM-1 Kinase inhibition for the most potent derivatives **7h** and **8f** demonstrated persuasive results as shown in (Table 2**, **Fig. 5). Specifically, the 3-cyanopyridine hybrid, **8f**, with methoxy group at position 2 and 2-naphthyl moiety at position 6, exhibited a comparable IC_50_ value of 0.58 µM, while the 3-cyanopyridin-2-one hybrid **7h**, with 3-pyridyl moiety at position 6, displayed the highest PIM-1 kinase inhibitory activity among all, with almost equipotent potency (IC_50_ = 0.283 µM) compared to the reference drug Staurosporine, (IC_50_ = 0.223 µM).

**Table 2:** The IC_50_ (µM) of PIM1 Kinase inhibition assays of the target cyanopyridine derivatives **7h** and **9f**

Ser	Compound		PIM1-Kinase
	ID	MW	IC_50_ (µM)
**1**	7h	452.46	0.281 ± 0.012
2	8f	515.55	0.58 ± 0.025
***	Staurosporine	466.54	0.223 ± 0.01

Thus, it can be inferred that the 3-cyanopyridine nucleus with such promising anti-breast cancer results of the synthesized target compounds, in particular, compound **7h**, still represents a significant scaffold in the quest for highly potent PIM-1 kinase inhibitors, which merits more investigation and SAR study.

##### Flow cytometric cell cycle analysis:

**Fig. 6:**
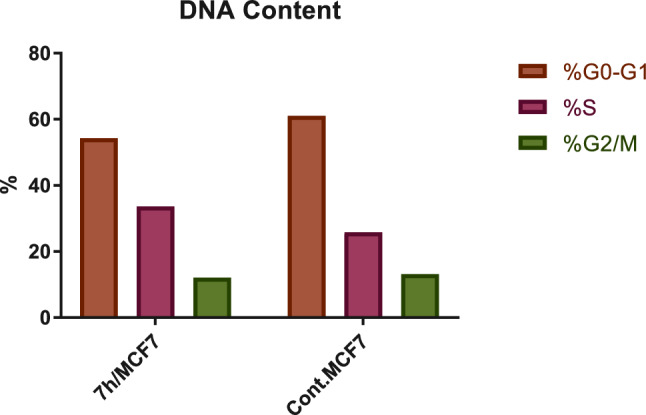
Cell cycle analysis in the MCF-7 cell line treated with compound **7h** compared with the control

The cell cycle encompasses four distinct phases: G1 phase, S phase (synthesis), G2 phase, and M phase. During G1, cellular amplification and preparation for DNA replication take place. The S phase involves DNA replication and chromatid duplication. In the G2 phase, DNA repair and further growth occur. The M phase is characterized by nuclear division. The investigation of the impact of compound **7h** on cell cycle progression and the induction of apoptosis in MCF-7 cells. The MCF-7 cell line was subjected to incubation with the IC_50_ concentration of compound **7h** for 24 h. Subsequently, the cells were stained with PI/Annexin V and analyzed using flow cytometry (BD FASCCalibur). Upon analyzing the results (Table 3, Fig. 6), it was evident that the percentage of cell accumulation in the S phase increased in MCF-7 cells treated with compound **7h** after 24 h of incubation. This observation indicates a cell cycle arrest at the S phase, with the percentage of cells accumulated in this phase rising from 25.83% in the control group to 33.61%.

**Table 3:** Cell cycle analysis t in MCF-7 cell line treated with **7h** compound

Ser	Sample		DNA content			
	Code	IC_50_ uM	%G0-G1	%S	%G2/M	Comment
1	7 h/MCF7	–	54.28	33.61	12.11	Cell growth arrest@ S
2	Cont.MCF7	–	61.03	25.83	13.14	–

**Fig. 7:**
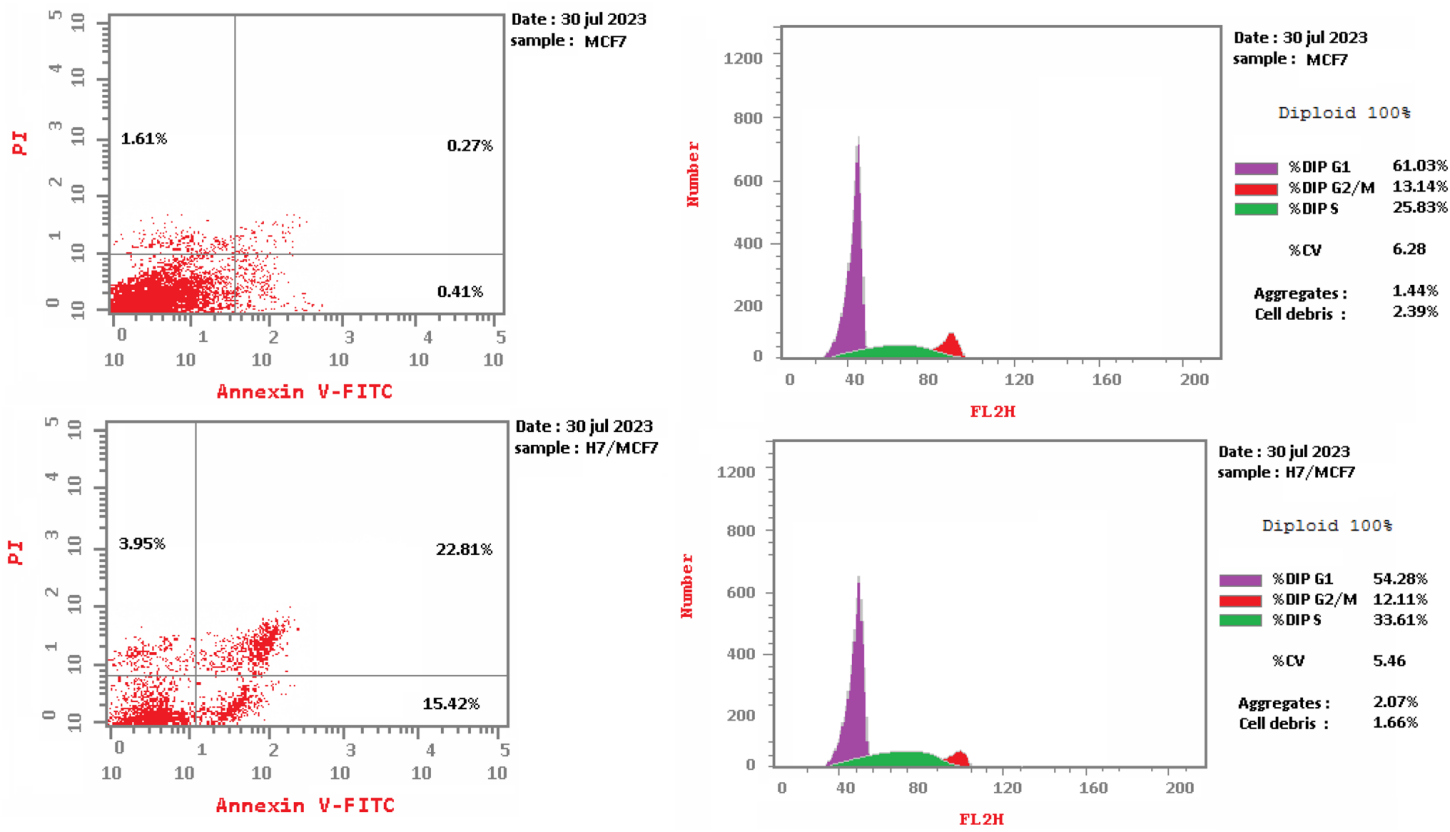
Cell cycle analysis and Apoptosis induction analysis using Annexin V/PI of compound **7h** and control untreated RPMI8226 cell

Compound **7h** was chosen to investigate its abilities in inducing cancer cell apoptosis. Cell cycle analysis of MCF-7 after treatment with compound **7h**. To corroborate the ability of the compound to induce apoptosis, cells were stained with Annexin V/PI, incubated for 24 h, and analyzed. Analysis of early and late apoptosis showed that compound **7h** was positively able to make significant levels of apoptosis with necrosis percent 3.95 (Table 4, Fig. 7).

**Table 4:** Cell cycle analysis and apoptosis detection of compound **7h**

Ser	Code	Conc	Apoptosis			Necrosis
			Total	Early	Late	
1	7 h/MCF7	–	42.18	15.42	22.81	3.95
2	Cont.MCF7	–	2.29	0.41	0.27	1.61

##### Molecular docking studies:

**Fig. 8:**
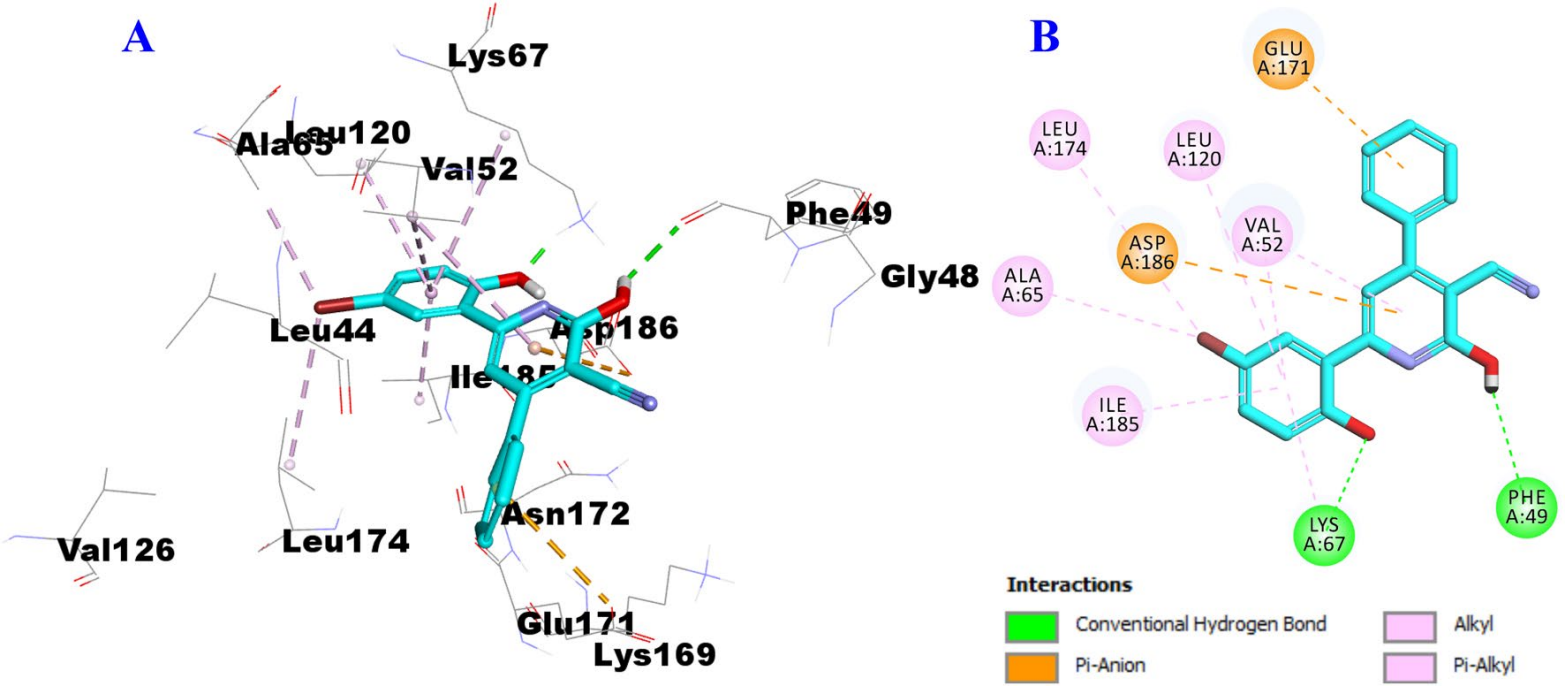
Docking and binding mode of VRV400 into the active site of PIM-1 (PDB entry: 2OBJ) (**A**) 3D structure of VRV400 (cyan) (**B**) 2D structure of VRV400 (cyan)

To study the ability of compounds **7h** and **8f** to bind to the PIM-1 kinase enzyme (PDB entry: 2OBJ), the ligand VRV40 was firstly docked. As illustrated in Fig. 8, ligand VRV400 engaged in the formation of two hydrogen bonds with Phe49 and Lys67 and many hydrophobic interactions (pi-cation, alkyl, and pi-alkyl) with Val52, Ala65, Lys67, Ile104, Leu120, Leu174, Ile185, and Asp186 amino acid residues.

**Fig. 9:**
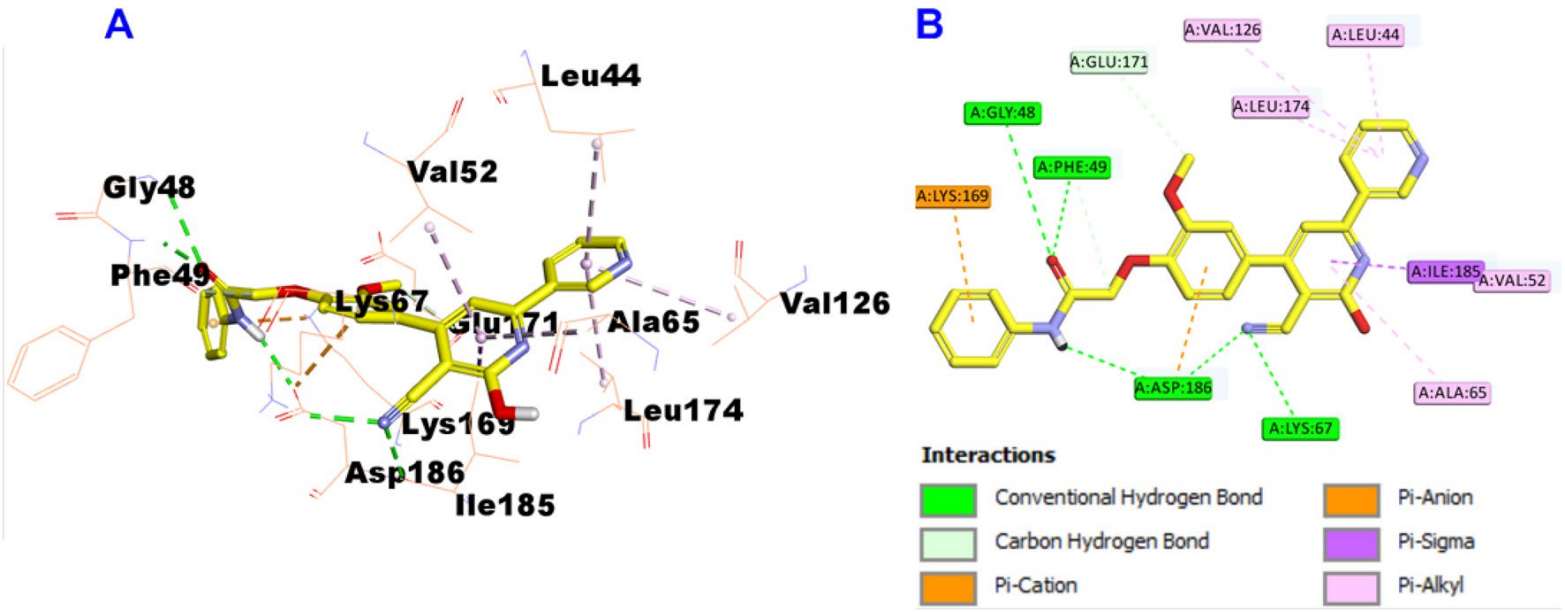
Docking and binding mode of hybrid **7h** into the active site of PIM-1 (PDB entry: 2OBJ) (**A**) 3D structure of hybrid **7h** (blue) (**B**) 2D structure of hybrid **7h** (blue)

Additionally, docking results of compound **7h** into the PIM-1 active site evidenced that compound **7h** displayed affinity of -10.1 kcal/mol and incorporated in the formation of five hydrogen bonds with Gly48, Phe49, Asp186 (two hydrogen bonds), and Lys67, in addition to numerous hydrophobic interactions such as van der Waals, pi-cation, pi-anion, pi-sigma, and pi-alkyl with Leu44, Phe49, Val52, Ala65, Val126, Lys169, Glu171, Leu174, Ile185, and Asp186 amino acid residues, which reflects its potency on inhibition of PIM-1 kinase enzymes and explains the highest potency of compound **7h** against the MCF7 breast cancer cell line, which may be attributed mechanistically to the activity of PIM-1 inhibitory activity (Fig. 9).

**Fig. 10:**
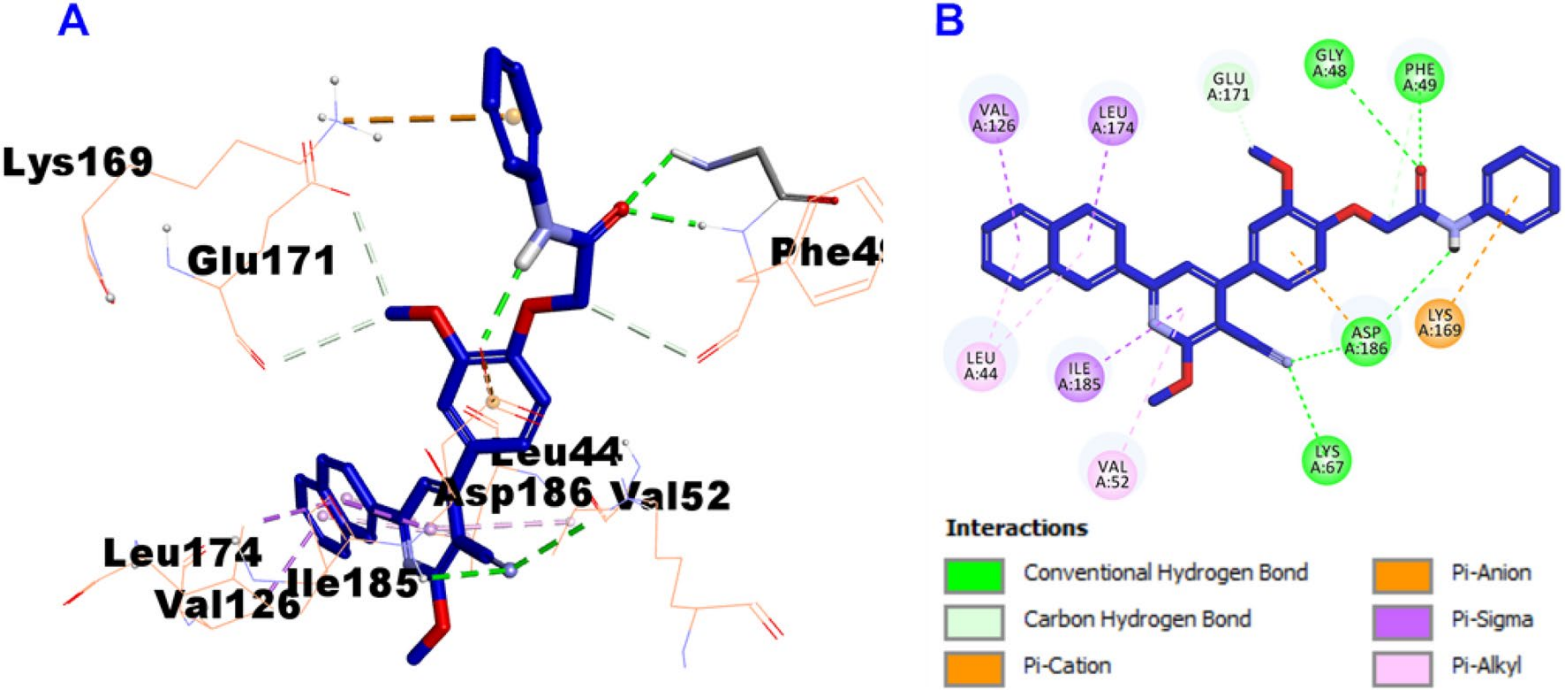
Docking and binding mode of compound **8f** into the active site of PIM-1 (PDB entry: 2OBJ) (**A**) 3D structure of compound **8f** (violet); **B** 2D structure of compound **8f** (violet)

Furthermore, docking results of compound **8f** into the PIM-1 active site proved that compound **8f** showed affinity of -9.9 kcal/mol and involved in the formation of five hydrogen bonds with Gly48, Phe49, Asp186 (two hydrogen bonds), and Lys67, in addition to several hydrophobic interactions such as carbon hydrogen bond, pi-cation, pi-anion, pi-sigma, and pi-alkyl with Leu44, Phe49, Val52, Val126, Lys169, Glu171, Leu174, Ile185, and Asp186 amino acid residues, which reflects its potency on inhibition of the PIM-1 kinase enzymes and explains the highest potency of compound **8f** against the MCF7 breast cancer cell line, which may be attributed mechanistically to the activity of PIM-1 inhibitor (Fig. 10).

Taken together, the docking results were in agreement with the biological study, and it could be concluded that compounds **7h** and **8f** are entitled to be used as a future template, which deserve further study, which were rapidly identified as an attractive lead candidate for PIM-1 inhibitor hybrids and were extensively profiled.

## Conclusion

In summary, the new derivatives of cyanopyridine **7a-j**, **8a-g**, **9a-g**, and **10** have been designed and synthesized utilizing one-pot multicomponent reaction (MCR). Most of the tested compounds displayed good anti-breast cancer (MCF-7) activity; in particular, compounds **7h**, and **8f** showed potent anticancer activity against MCF-7 *via* inhibition of PIM-1 kinase. Moreover, compound **7h** arrested the tumor cells at the S phase. In addition, compound **7h** caused cell death mainly by inducing early and late apoptosis. Taken together, the molecular docking study of **7h** and **8f**, along with the biological screening could be concluded that the *N*- and *O*-alkyl cyanopyridine scaffold is considered a promising scaffold for innovation of potent anticancer candidates, and it merits more investigation and SAR study, which are ongoing in our laboratory.

## Experimental

### Chemistry

#### General information

All information about reagents and spectral analyses were showed in Supporting Information.

##### Synthesis of Sodium 4-formyl-2-methoxyphenolate (2):

To a solution of sodium ethoxide (0.075 g, 3.0 mmol) in 30 mL of ethanol, vanillin (0.5 g, 3.0 mmol) was added portion-wise with stirring in an ice bath for 1h. The solvent was evaporated under vacuum, and the formed precipitate was collected, washed with ether, dried, and used as commercial material. White powder, yield: 0.57 g (99%); mp. > 340 °C; FT-IR (ATR) *v*_*max*_: 3052 (CH_arom._), 2971, 2936 (CH_aliph._), 1669 (C = O_formyl_) cm^−1^; ^1^HNMR (400 MHz, DMSO-*d*_*6*_) *δ:* 9.31 (s, 1H, CH_formyl_), 7.15 (d, 1H, *J* = 7.9 Hz, CH_arom._), 7.02 (s, 1H, CH_arom._), 6.31(d, 1H, *J* = 7.9 Hz, CH_arom._), 3.71 (s, 3H, OCH_3_). ^13^C NMR (100 MHz, DMSO-*d*_*6*_) *δ:* 187.1 (C = O_formyl_), 170.6, 151.7, 130.9, 118.7, 117.8, 108.0, 55.1 (OCH_3_) ppm. *Anal*. Calcd. for C_8_H_7_NaO_3_ (174.13): C, 55.18; H, 4.05%. Found: C, 55.30; H, 4.18%.

##### 3-Methoxy-4-(2-oxo-2-phenylethoxy)benzaldehyde (3):

White crystal, yield 0.80 g (98%), mp. 154–156 ^o^C, was prepared according to literature procedure [38].

##### Procedure for synthesis of ethyl-3-[4-(2-anilino-2-oxoethoxy)-3-methoxyphenyl]-2-cyanoacrylate (4):

A mixture of compound **3** (0.5 g, 1.75 mmol) and respective active methylene compounds such as: ethyl cyanoacetate (0.19 g, 1.75 mmol) were refluxed in the presence of TEA in 20 mL ethanol for 1h. The formed precipitate was filtered off on hot, washed with ethanol, and crystallized from acetonitrile. Yellow powder, yield: 0.63 g (94%), mp. 138–140 °C; FT-IR (ATR) *v*_*max*_: 3389 (NH), 3062 (CH_arom._), 2983, 2919, 2866 (CH_aliph._), 2223 (CN); 1714 (C = O_ester_); 1691 (C = O_amide_) cm^−1^; ^1^H NMR (400 MHz, DMSO-*d*_*6*_) *δ:* 10.22 (s, 1H, NH), 8.33 (s, 1H, CH_olefinic_), 7.84 (d, 1H,* J* = 1.5 Hz, CH_arom._), 7.73 (d, 1H,* J* = 8.5 Hz, CH_arom._), 7.62 (d, 2H,* J* = 7.9 Hz, CH_arom._), 7.34 (t, 2H, *J* = 7.8 Hz, CH_arom_), 7.15 (d, 1H, *J* = 8.5 Hz, CH_arom._), 7.09 (t, 1H,* J* = 7.3 Hz, CH_arom._), 4.89 (s, 2H, OCH_2_CO), 4.32 (q, 2H, *J* = 7.1 Hz, OCH_2_CH_3_), 3.87 (s, 3H, OCH_3_); 1.32 (t, 3H, *J* = 7.1 Hz, OCH_2_CH_3_). *Anal*. Calcd. for C_21_H_20_N_2_O_5_ (380.39): C, 66.31; H, 5.30; N, 7.36%. Found: C, 66.45; H, 5.19; N, 7.22%.

##### General procedure for synthesis of pyridines 7a-j:

To a solution of arylidene **4** (0.5 g, 1.3 mmol) and ammonium acetate **5** (0.51 g, 6.5 mmol) in 30 mL of ethanol, 1.3 mmol of respective methylaryl/heterylketone derivatives **6a-j** such as acetophenone (0.16 g), 3-methoxyacetophenone (0.2 g), 4-methoxyacetophenone (0.2 g), 4-chloroacetophenone (0.2 g), 4-bromoacetophenone (0.26 g), 2-acetylfuran (0.14 g), 2-acetylthiophene (0.17 g), 3-acetylpyidine (0.16 g), 4-acetylpyridine (0.16 g) and/or 2-acetyl naphthalene (0.22 g) was added. The reaction mixture was refluxed for 6–8 h. (monitored using TLC). The formed precipitate was filtered off (on hot), washed with water several times, then with ethanol, dried, and crystallized from a mixture of ethanol and DMF (1:1).

##### 2-(4-(3-Cyano-2-oxo-6-phenyl-1,2-dihydropyridin-4-yl)-2-methoxyphenoxy)-*N*phenylacetamide(7a):

Pale yellow powder, yield: 0.53 g (89%), mp. 276–278 °C; FT-IR (ATR) *v*_*max*_: 3390 (NH), 3076 (CH_arom._), 2969, 2917 (CH_aliph._), 2217 (CN), 1690 (C = O_amide_) cm^−1^; ^1^H NMR (400 MHz, DMSO-*d*_*6*_) *δ:* 12.62 (s, 1H, NH), 10.02 (s, 1H, NH), 7.90 (d, 2H,* J* = 6.8 Hz, CH_arom._), 7.63 (d, 2H,* J* = 7.8 Hz, CH_arom._), 7.57–7.53 (m, 3H, CH_arom._), 7.42 (s, 1H, CH_arom._), 7.34 (t, 3H,* J* = 7.3 Hz, CH_arom._), 7.15 (d, 1H, *J* = 8.4 Hz, CH_arom._), 7.09 (t, *J* = 7.3 Hz, 1H, CH_arom._), 6.86 (s, 1H, CH_arom._), 4.81 (s, 2H, CH_2_); 3.92 (s, 3H, OCH_3_). ^13^C NMR (100 MHz, DMSO-*d*_*6*_) *δ*: 166.7, 162.6, 159.8, 151.7, 149.8, 149.4, 138.7, 132.9, 131.5, 129.8, 129.3, 129.2, 128.1, 124.2, 121.8, 120.0, 117.2, 114.4, 113.1, 106.6, 90.3, 68.6 (CH_2_), 56.4 (OCH_3_). *Anal*. Calcd. for C_27_H_21_N_3_O_4_ (451.47): C, 71.83; H, 4.69; N, 9.31%. Found: C, 71.61; H, 4.81; N, 9.50%.

##### 2-(4-(3-Cyano-6-(3-methoxyphenyl)-2-oxo-1,2-dihydropyridin-4-yl)-2methoxyphenoxy)-*N*-phenylacetamide (7b):

Pale yellow powder, yield: 0.55 g (87%), mp. 244–246 °C; FT-IR (ATR) *v*_*max*_:3378 (NH), 3065 (CH_arom._), 2930, 2842 (CH_aliph._)_._, 2220 (CN), 1690 (C = O_amide_) cm^−1^; ^1^H NMR (400 MHz, DMSO-*d*_*6*_) *δ:* 12.66 (s, 1H, NH), 10.09 (s, 1H, NH), 7.65 (d, 2H,* J* = 7.7 Hz, CH_arom._), 7.47–7.42 (m, 4H, CH_arom._), 7.36–7.32 (m, 3H, CH_arom._), 7.15–7.08 (m, 3H, CH_arom._), 6.88 (s, 1H, CH_arom._), 4.82 (s, 2H, CH_2_), 3.92 (s, 3H, OCH_3_), 3.86 (s, 3H, OCH_3_), ^13^C NMR (100 MHz, DMSO-*d*_*6*_) *δ:* 166.7, 162.5, 159.9, 159.8, 151.2, 149.8, 149.3, 138.8, 134.1, 130.5, 129.7, 129.2, 124.1, 121.8, 120.4, 119.9, 117.7, 117.2, 114.1, 113.1, 106.5, 98.4, 68.5 (CH_2_), 56.4 (OCH_3_), 55.8 (OCH_3_). *Anal*. Calcd. for C_28_H_23_N_3_O_5_ (481.49): C, 69.84; H, 4.81; N, 8.73%. Found: C, 69.96; H, 4.67; N, 8.43%.

##### 2-(4-(3-Cyano-6-(4-methoxyphenyl)-2-oxo-1,2-dihydropyridin-4-yl)-2-methoxyphenoxy)-*N*-phenylacetamide (7c):

Pale yellow powder, yield: 0.56 g (89%), mp. 284–286 °C; FT-IR (ATR) *v*_*max*_:3336 (NH), 3079 (CH_arom._), 2936, 2837 (CH_aliph._)_._, 2213 (CN), 1691 (C = O_amide_) cm^−1^; ^1^H NMR (400 MHz, DMSO-*d*_*6*_) *δ:* 12.44 (s, 1H, NH), 10.00 (s, 1H, NH), 7.90 (s, 2H, CH_arom._), 7.64 (s, 2H, CH_arom._), 7.41–7.33 (m, 4H, CH_arom._), 7.15–7.09 (m, 4H, CH_arom._), 6.80 (s, 1H, CH_arom._), 4.81 (s, 2H, CH_2_), 3.93 (s, 3H, OCH_3_); 3.86 (s, 3H, OCH_3_). ^13^C NMR (100 MHz, DMSO-*d*_*6*_) *δ:* 166.7, 162.7, 162.1, 159.8, 149.6, 149.2, 138.6, 129.8, 129.7, 129.3, 124.6, 124.3, 121.7, 119.9, 117.5, 114.8, 113.8, 112.7, 105.6, 105.5, 97.1, 68.2 (CH_2_), 56.3 (OCH_3_), 55.9 (OCH_3_)*. Anal*. Calcd. for C_28_H_23_N_3_O_5_ (481.49): C, 69.84; H, 4.81; N, 8.73%. Found: C, 69.59; H, 4.63; N, 8.89%.

##### 2-(4-(6-(4-Chlorophenyl)-3-cyano-2-oxo-1,2-dihydropyridin-4-yl)-2methoxyphenoxy)-*N*-phenylacetamide (7d):

Pale yellow powder, yield: 0.53 g (85%), mp. 276–278 °C; FT-IR (ATR) *v*_*max*_: 3380 (NH), 3083 (CH_arom._), 2926, 2839 (CH_aliph._), 2219 (CN), 1686 (C = O_amide_) cm^−1^; ^1^H NMR (400 MHz, DMSO-*d*_*6*_) *δ:* 12.74 (s, 1H, NH), 10.15 (s, 1H, NH), 7.92 (s, 2H, CH_arom._), 7.61 (s, 4H, CH_arom._), 7.40–7.33 (m, 4H, CH_arom._), 7.10 (s, 2H, CH_arom._), 6.91 (s, 1H, CH_arom._), 4.81 (s, 2H, CH_2_); 3.90 (s, 3H, OCH_3_). ^13^C NMR (100 MHz, DMSO-*d*_*6*_) *δ*: 166.7, 165.5, 158.6, 149.9, 149.7, 149.4, 138.7, 136.4, 130.0, 129.7, 129.5, 129.3, 129.2, 128.9, 124.2, 121.8, 120.0, 114.4, 113.7, 113.1, 93.8, 68.6 (CH_2_), 56.4 (OCH_3_). *Anal*. Calcd. for C_27_H_20_ClN_3_O_4_ (485.91): C, 66.74; H, 4.15; N, 8.65%. Found: C, 66.56; H, 4.35; N, 8.43%.

##### 2-(4-(6-(4-Bromophenyl)-3-cyano-2-oxo-1,2-dihydropyridin-4-yl)-2-methoxy phenoxy)-*N*-phenylacetamide (7e):

Pale yellow powder, yield: 0.6 g (86%), mp. 329–331 °C; FT-IR (ATR) *v*_*max*_: 3381 (NH), 3060 (CH_arom._), 2924, 2838 (CH_aliph._), 2216 (CN), 1689 (C = O_amide_) cm^−1^; ^1^H NMR (400 MHz, DMSO-*d*_*6*_) *δ:* 12.63 (s, 1H, NH), 10.02 (s,1H, NH), 7.87 (d, 2H,* J* = 8.0 Hz, CH_arom._), 7.74 (d, 2H,* J* = 8.2 Hz, CH_arom._), 7.64 (d, 2H,* J* = 7.9 Hz, CH_arom._), 7.42 (s, 1H, CH_arom._), 7.36–7.32 (m, 3H, CH_arom_), 7.15 (d, 1H,* J* = 8.4 Hz, CH_arom._), 7.09 (t, 1H, *J* = 7.3 Hz, CH_arom._), 6.92 (s, 1H, CH_arom._), 4.81 (s, 2H, CH_2_), 3.92 (s, 3H, OCH_3_). ^13^C NMR (100 MHz, DMSO-*d*_*6*_) *δ:* 166.7, 166.2, 163.1, 158.8, 155.6, 149.8, 149.3, 142.3, 138.8, 132.3, 130.2, 129.8, 129.6, 129.2, 125.2, 124.1, 121.8, 119.9, 114.1, 113.0, 93.9, 68.5 (CH_2_), 56.4 (OCH_3_). *Anal*. Calcd. for C_27_H_20_BrN_3_O_4_ (530.37): C, 61.14; H, 3.80; N, 7.92%. Found: C, 61.35; H, 3.63; N, 7.71%.

##### 2-(4-(3-Cyano-6-(furan-2-yl)-2-oxo-1,2-dihydropyridin-4-yl)-2-methoxyphenoxy)-*N*-phenylacetamide (7f):

Pale yellow powder, yield: 0.53 g (91%), mp. 296–298 ^o^C; FT-IR (ATR) *v*_*max*_: 3398 (NH), 3020 (CH_arom._), 2930, 2810 (CH_aliph._), 2219 (CN), 1636 (C = O_amide_) cm^−1^; ^1^H NMR (400 MHz, DMSO-*d*_*6*_) *δ:* 12.70 (s, 1H, NH), 10.09 (s,1H, NH), 8.00 (s, 1H, CH_arom._), 7.65–7.63 (m, 3H, CH_arom._), 7.38–7.28 (m, 4H, CH_arom._), 7.14–7.09 (m, 2H, CH_arom._), 6.84 (s, 1H, CH_arom._), 6.78 (s, 1H, CH_arom._), 4.82 (s, 2H, CH_2_), 3.91 (s, 3H, OCH_3_). ^13^C NMR (100 MHz, DMSO-*d*_*6*_) *δ:* 166.7, 162.1, 159.7, 149.8, 149.3, 147.3, 146.1, 140.8, 138.7, 129.6, 129.2, 124.2, 121.6, 120.0, 117.2, 114.7, 114.1, 113.6, 112.7, 103.1, 97.6, 68.4 (CH_2_), 56.3 (OCH_3_). *Anal*. Calcd. for C_25_H_19_N_3_O_5_ (441.43): C, 68.02; H, 4.34; N, 9.52%. Found: C, 68.23; H, 4.19; N, 9.74%.

##### 2-(4-(3-Cyano-2-oxo-6-(thiophen-2-yl)-1,2-dihydropyridin-4-yl)-2-methoxyphenoxy)-*N*-phenylacetamide (7 g):

Pale yellow powder, yield: 0.54 g (90%), mp. 293–295 °C; FT-IR (ATR) *v*_*max*_:3399 (NH), 3030 (CH_arom._), 2944, 2822 (CH_aliph._), 2221(CN), 1638 (C = O_amide_) cm^−1^; ^1^H NMR (400 MHz, DMSO-*d*_*6*_) *δ:* 12.76 (s, 1H, NH), 10.18 (s, 1H, NH), 8.06 (d, 1H, *J* = 3.7 Hz, CH_arom._), 7.87 (d, 1H, *J* = 3.8 Hz, CH_arom._), 7.65 (d, 2H, *J* = 7.7 Hz, CH_arom._), 7.37–7.24 (m, 6H, CH_arom._), 7.13–7.07 (m, 2H, CH_arom._), 4.83 (s, 2H, CH_2_), 3.91 (s, 3H, OCH_3_). ^13^C NMR (100 MHz, DMSO-*d*_*6*_) *δ:* 166.8, 163.2, 158.8, 149.6, 149.4, 149.3, 147.3, 138.5, 131.7, 129.9, 129.6, 129.4, 129.3, 124.4, 121.6, 120.1, 117.1, 114.2, 112.8, 106.8, 99.8, 68.4 (CH_2_), 56.3 (OCH_3_). *Anal*. Calcd. for C_25_H_19_N_3_O_4_S (457.50): C, 65.63; H, 4.19; N, 9.18%. Found: C, 65.39; H, 4.36; N, 9.40%.

##### 2-(4-(3-Cyano-2-oxo-6-(pyridin-3-yl)-1,2-dihydropyridin-4-yl)-2-methoxyphenoxy)-*N-*phenylacetamide (7 h):

Pale yellow powder, yield: 0.51 g (85%), mp. 295–297 °C; FT-IR (ATR) *v*_*max*_:3388 (NH), 3043 (CH_arom._), 2935, 2848 (CH_aliph._), 2218 (CN), 1682 (C = O_amide_) cm^−1^; ^1^H NMR (400 MHz, DMSO-*d*_*6*_) *δ:* 12.90 (s, 1H, NH), 10.18 (s,1H, NH), 9.10 (s,1H, CH_arom._), 8.73 (d, 1H, *J* = 3.9 Hz, CH_arom._), 8.30 (d, 1H, *J* = 7.5 Hz, CH_arom._), 7.65 (d, 2H, *J* = 7.7 Hz, CH_arom._), 7.59–7.56 (m, 1H, CH_arom._), 7.44 (s, 1H, CH_arom._), 7.39–7.33 (m, 3H, CH_arom._), 7.14–7.08 (m, 2H, CH_arom._), 7.02 (s, 1H, CH_arom._), 4.84 (s, 2H, CH_2_), 3.92 (s, 3H, OCH_3_). ^13^C NMR (100 MHz, DMSO-*d*_*6*_) *δ:* 166.7, 162.7, 159.6, 151.9, 149.8, 149.3, 148.9, 138.8, 135.8, 129.3, 129.2, 129.1, 129.1, 124.2, 124.1, 121.9, 119.9, 117.2, 113.8, 112.9, 107.5, 98.7, 68.3 (CH_2_), 56.3 (OCH_3_)*. Anal*. Calcd. for C_26_H_20_N_4_O_4_ (452.46): C, 69.02; H, 4.46; N, 12.38%. Found: C, 69.29; H, 4.70; N, 12.15%.

##### 2-(4-(3-Cyano-2-oxo-6-(pyridin-4-yl)-1,2-dihydropyridin-4-yl)-2-methoxyphenoxy)-*N*-phenylacetamide (7i):

Pale yellow powder, yield: 0.52 g (87%), mp. 280–282 °C; FT-IR (ATR) *v*_*max*_:3388 (NH), 3050 (CH_arom._), 2925, 2830 (CH_aliph._), 2218(CN), 1682 (C = O_amide_) cm^−1^; ^1^H NMR (400 MHz, DMSO-*d*_*6*_) *δ:* 12.79 (s, 1H, NH), 10.03 (s,1H, NH), 8.75 (d, 2H, *J* = 5.0 Hz, CH_arom._), 7.91 (d, 2H, *J* = 4.3 Hz, CH_arom._), 7.64 (d, 2H, *J* = 7.7 Hz, CH_arom._), 7.44 (s, 1H, CH_arom._), 7.37–7.32 (m, 3H, CH_arom._), 7.17–7.08 (m, 3H, CH_arom._), 4.82 (s, 2H, CH_2_), 3.93 (s, 3H, OCH_3_). ^13^C NMR (100 MHz, DMSO-*d*_*6*_) *δ:* 166.8, 162.8, 159.6, 150.6, 149.7, 149.5, 149.4, 140.6, 138.3, 129.4, 129.3, 124.5, 122.1, 121.9, 120.2, 116.8, 114.3, 113.0, 108.3, 99.3, 68.4 (CH_2_), 56.4 (OCH_3_). *Anal*. Calcd. for C_26_H_20_N_4_O_4_ (452.46): C, 69.02; H, 4.46; N, 12.38%. Found: C, 68.18; H, 4.25; N, 12.54%.

##### 2-(4-(3-Cyano-6-(naphthalen-2-yl)-2-oxo-1,2-dihydropyridin-4-yl)-2-methoxyphenoxy)-*N*-phenylacetamide (7j):

Pale yellow powder, yield: 0.58 g (88%), mp. > 300 °C; FT-IR (ATR) *v*_*max*_:3388 (NH), 3045 (CH_arom._), 2938, 2817 (CH_aliph._), 2217 (CN), 1690 (C = O_amide_) cm^−1^; ^1^H NMR (400 MHz, DMSO-*d*_*6*_) *δ:* 12.85 (s, 1H, NH), 10.20 (s, 1H, NH), 8.55 (s, 1H, CH_arom._), 8.05–8.01 (m, 4H, CH_arom._), 7.66 (s, 4H, CH_arom._), 7.46–7.35 (m, 4H, CH_arom._), 7.15–7.04 (m, 3H, CH_arom._), 4.85 (s, 2H, CH_2_), 3.93 (s, 3H, OCH_3_). *Anal*. Calcd. for C_31_H_23_N_3_O_4_ (501.53): C, 74.24; H, 4.62; N, 8.38%. Found: C, 74.51; H, 4.39; N, 8.56%.

##### General procedure for synthesis of *O*-methylcyanopyridines 8a-g, *N*-methylcyanopyridines 9a-g and bipyridinium iodide salt 10:

To a stirred mixture of pyridines **7a-f**, **7h**, and **7j** (1.1 mmol) and potassium carbonate (0.15 g, 1.1 mmol) in 20 mL of DMF, methyl iodide (0.16 g, 1.2 mmol) was added drop by drop in about half an hour at room temperature. The reaction mixture was continuously stirred overnight and then poured into 50 mL of water. The formed precipitate (two products except compound **10**) was filtered off, washed with water, dried, and separated by thin layer chromatography using a mixture of chloroform and petroleum ether (9:1) (Rf_**8a-g**_ = 0.17, 0.16, 0.20, 0.18, 0.17, 0.19, and 0.10, respectively; Rf_**9a-g**_ = 0.41, 0.32, 0.47, 0.43, 0.23, 0.32, and 0.27, respectively; and Rf_**10**_ = 0.11).

##### 2-(4-(3-Cyano-2-oxo-6-phenyl-1,2-dihydropyridin-4-yl)-2-methoxyphenoxy)-*N*-phenylacetamide (8a):

White powder, yield: 0.23 g (45%), mp. 194–196 °C; FT-IR (ATR) *v*_*max*_: 3393 (NH), 3065 (CH_arom._), 2944, 2851 (CH_aliph._), 2217 (CN), 1691 (C = O_amide_) cm^−1^; ^1^H NMR (400 MHz, DMSO-*d*_*6*_) *δ*: 10.10 (s, 1H, NH), 8.27 (d, 2H,* J* = 3.7 Hz, CH_arom._), 7.83 (s, 1H, CH_arom._), 7.65 (d, 2H,* J* = 7.5 Hz, CH_arom._), 7.54 (s, 3H, CH_arom._), 7.44 (s, 1H, CH_arom._), 7.34 (s, 3H, CH_arom._), 7.16 (d, 1H, *J* = 8.2 Hz, CH_arom._), 7.09 (t, 1H, *J* = 6.5 Hz, CH_arom._), 4.82 (s, 2H, CH_2_), 4.16 (s, 3H, OCH_3_), 3.93 (s, 3H, OCH_3_). ^13^C NMR (100 MHz, DMSO-*d*_*6*_) *δ*: 166.7, 164.9, 157.5, 156.4, 149.5, 149.4, 138.8, 137.2, 131.1, 129.4, 129.3, 129.2, 127.9, 124.1, 121.9, 119.8, 116.1, 114.2, 114.1, 113.2, 92.4, 68.4 (CH_2_), 56.3 (OCH_3_), 54.96 (OCH_3_). *Anal*. Calcd. for C_28_H_23_N_3_O_4_ (465.49): C, 72.24; H, 4.98; N, 9.03%. Found: C, 72.43; H, 4.79; N, 9.19%.

##### 2-(4-(3-Cyano-2-methoxy-6-(3-methoxyphenyl)-pyridin-4-yl)-2-methoxy-phenoxy)-*N*-phenylacetamide (8b):

White powder, yield: 0.21 g (40%), mp. 158–160 °C; FT-IR (ATR) *v*_*max*_: 3396 (NH), 3067 (CH_arom._), 2944, 2911, 2843 (CH_aliph._), 2218 (CN), 1693 (C = O_amide_) cm^−1^; ^1^H NMR (400 MHz, DMSO-*d*_*6*_) *δ*: 10.13 (s, 1H, NH), 7.82–7.78 (m, 3H, CH_arom._), 7.65 (d, 2H,* J* = 4.2 Hz, CH_arom._), 7.44 (s, 2H, CH_arom._), 7.34 (s, 3H, CH_arom._), 7.16–7.10 (m, 3H, CH_arom._), 4.83 (s, 2H, CH_2_), 4.15 (s, 3H, OCH_3_), 3.93 (s, 3H, OCH_3_), 3.86 (s, 3H, OCH_3_). ^13^C NMR (100 MHz, DMSO-*d*_*6*_) *δ:* 166.7, 164.7, 160.1, 157.3, 156.4, 149.5, 149.0, 149.4, 138.8, 138.6, 130.4, 129.4, 129.2, 124.1, 121.9, 120.3, 119.9, 116.5, 116.1, 114.2, 114.1, 113.3, 92.5, 68.4 (CH_2_), 56.3 (OCH_3_), 55.7 (OCH_3_), 54.9 (OCH_3_). *Anal*. Calcd. for C_29_H_25_N_3_O_5_ (495.52): C, 70.29; H, 5.09; N, 8.48%. Found: C, 70.51; H, 5.22; N, 8.36%.

##### 2-(4-(3-Cyano-2-methoxy-6-(4-methoxyphenyl)pyridin-4-yl)-2-methoxyphenoxy)-*N*-phenylacetamide (8c):

White powder, yield: 0.21 g (40%), mp. 188–190 °C; FT-IR (ATR) *v*_*max*_: 3398 (NH), 3070 (CH_arom._), 2944, 2914, 2850 (CH_aliph._), 2222 (CN), 1693 (C = O_amide_) cm^−1^; ^1^H NMR (400 MHz, DMSO-*d*_*6*_) *δ*: 10.10 (s, 1H, NH), 8.27 (s, 2H, CH_arom._), 7.82–7.55 (m, 6H, CH_arom._), 7.35 (s, 3H, CH_arom._), 7.15 (s, 2H, CH_arom._), 4.82 (s, 2H, CH_2_), 4.16 (s, 3H, OCH_3_), 3.96 (s, 3H, OCH_3_), 3.93 (s, 3H, OCH_3_). ^13^C NMR (100 MHz, DMSO-*d*_*6*_) *δ*: 166.7, 164.8, 161.9, 157.6, 156.4, 156.2, 149.5, 138.8, 131.0, 129.6, 129.2, 127.9, 124.1, 121.9, 121.8, 119.9, 114.7, 114.4, 114.0, 113.4, 92.4, 68.6 (CH_2_), 56.4 (OCH_3_), 55.8 (OCH_3_), 54.9 (OCH_3_). *Anal*. Calcd. for C_29_H_25_N_3_O_5_ (495.52): C, 70.29; H, 5.09; N, 8.48%. Found: C, 70.13; H, 5.27; N, 8.63%.

##### 2-(4-(6-(4-Chlorophenyl)-3-cyano-2-oxo-1,2-dihydropyridin-4-yl)-2-methoxyphenoxy)-*N*-phenylacetamide (8d):

White powder, yield: 0.15 g (30%), mp. 243–245 °C; FT-IR (ATR) *v*_*max*_:3390 (NH), 3069 (CH_arom._), 2947, 2921, 2850 (CH_aliph._), 2218 (CN), 1689 (C = O_amide_) cm^−1^; ^1^H NMR (400 MHz, DMSO-*d*_*6*_) *δ*: 10.10 (s, 1H, NH), 8.30 (s, 2H, CH_arom._), 7.86 (s, 1H, CH_arom._), 7.64–7.60 (m, 4H, CH_arom._), 7.44 (s, 1H, CH_arom._), 7.34 (s, 3H, CH_arom._), 7.16 (d, 1H,* J* = 7.4 Hz, CH_arom._), 7.10 (s, 1H, CH_arom._), 4.82 (s, 2H, CH_2_), 4.15 (s, 3H, OCH_3_), 3.93 (s, 3H, OCH_3_); ^13^C NMR (100 MHz, DMSO-*d*_*6*_) *δ:* 166.7, 164.9, 156.6, 156.2, 149.6, 138.8, 136.1, 135.9, 129.7, 129.5, 129.3, 129.2, 124.1, 122.0, 120.0, 114.6, 114.3, 114.1, 113.6, 113.4, 92.8, 68.8 (CH_2_), 56.5 (OCH_3_), 55.0 (OCH_3_). *Anal*. Calcd. for C_28_H_22_ClN_3_O_4_ (499.94): C, 67.27; H, 4.44; N, 8.40%. Found: C, 67.45; H, 4.29; N, 8.58%.

##### 2-(4-(6-(4-Bromophenyl)-3-cyano-2-methoxypyridin-4-yl)-2-methoxyphenoxy)-*N*-phenylacetamide (8e):

White powder, yield: 0.154 g (30%), mp. 246–248 °C; FT-IR (ATR) *v*_*max*_:3386 (NH), 3060 (CH_arom._), 2994,2920, 2848 (CH_aliph._), 2218 (CN), 1692 (C = O_amide_) cm^−1^; ^1^H NMR (400 MHz, DMSO-*d*_*6*_) *δ*: 10.10 (s, 1H, NH), 8.21 (d, 2H,* J* = 7.2 Hz, CH_arom._), 7.84 (s, 1H, CH_arom._), 7.73 (d, 2H,* J* = 7.3 Hz, CH_arom._), 7.64 (d, 2H, *J* = 6.6 Hz, CH_arom._), 7.43 (s, 1H, CH_arom._), 7.34 (s, 3H, CH_arom._), 7.16–7.09 (m, 2H, CH_arom._), 4.82 (s, 2H, CH_2_), 4.14 (s, 3H, OCH_3_), 3.93 (s, 3H, OCH_3_); ^13^C NMR (100 MHz, DMSO-*d*_*6*_) *δ*: 166.7, 164.9, 156.6, 156.4, 149.7, 149.6, 138.8, 136.4, 132.3, 129.9, 129.5, 129.2, 124.8, 124.1, 122.0, 120.0, 115.9, 114.6, 114.1, 113.5, 92.9, 68.7 (CH_2_), 56.5 (OCH_3_), 55.0 (OCH_3_). *Anal*. Calcd. for C_28_H_22_BrN_3_O_4_ (544.39): C, 61.77; H, 4.07; N, 7.72%. Found: C, 61.54; H, 4.29; N, 7.50%.

##### 2-(4-(3-Cyano-6-(naphthalen-2-yl)-2-oxo-1,2-dihydropyridin-4-yl)-2-methoxy phenoxy)-*N*-phenylacetamide (8f):

Pale yellow powder, yield: 0.18 g (35%), mp. 193–195°C; FT-IR (ATR) *v*_*max*_: 3395 (NH), 3053 (CH_arom._), 2942, 2918, 2850 (CH_aliph._)_._, 2217 (CN), 1690 (C = O_amide_) cm^−1^; ^1^H NMR (400 MHz, DMSO-*d*_*6*_) *δ:* 10.10 (s, 1H, NH), 8.89 (s, 1H, CH_arom._), 8.41 (d, 1H, *J* = 8.5, CH_arom._), 8.10–8.07 (m, 2H, CH_arom._), 8.01–7.99 (m, 2H, CH_arom._), 7.66–7.60 (m, 4H, CH_arom._), 7.48 (s, 1H, CH_arom._), 7.39–7.33 (m, 3H, CH_arom._), 7.18 (d, 1H, *J* = 7.2, CH_arom._), 7.10 (t, 1H, *J* = 7.2, CH_arom._), 4.83 (s, 2H, CH_2_), 4.23 (s, 3H, OCH_3_), 3.95 (s, 3H, OCH_3_); ^13^C NMR (100 MHz, DMSO-*d*_*6*_) *δ:* 166.7, 164.9, 157.4, 156.5, 149.5, 149.4, 138.8, 134.7, 134.5, 134.3, 133.3, 129.5, 129.4, 129.3, 128.9, 128.2, 128.1, 127.2, 124.9, 124.1, 121.9, 119.8, 116.2, 114.4, 114.1, 113.3, 92.4, 68.4 (CH_2_), 56.3 (OCH_3_), 55.0 (OCH_3_). *Anal*. Calcd. for C_32_H_25_N_3_O_4_ (515.55): C, 74.55; H, 4.89; N, 8.15%. Found: C, 74.68; H, 4.75; N, 8.34%.

##### 2-(4-(3-Cyano-6-(furan-2-yl)-2-methoxypyridin-4-yl)-2-methoxyphenoxy)-*N*-phenyl acetamide (8 g):

Pale yellow powder, yield: 0.21 g (40%), mp. 178–180 °C; FT-IR (ATR) *v*_*max*_: 3387 (NH), 3061 (CH_arom._), 2949, 2912, 2846 (CH_aliph._), 2213 (CN), 1686 (C = O_amide_) cm^−1^; ^1^H NMR (400 MHz, CDCl_3_) *δ:* 8.71 (s, 1H, NH), 7.53 (d, 2H, *J* = 7.9 Hz, CH_arom._), 7.49 (s, 1H, CH_arom._), 7.32 (s, 1H, CH_arom._), 7.28 (t, 2H, *J* = 7.6 Hz, CH_arom._), 7.17 -7.13 (m, 3H, CH_arom._), 7.06 (t, 1H, *J* = 7.3 Hz, CH_arom._), 7.00 (d, 1H, *J* = 8.2 Hz, CH_arom._), 6.50 (s,1H, CH_arom._), 4.62 (s, 2H, CH_2_), 4.05 (s, 3H, OCH_3_), 3.94 (s, 3H, OCH_3_); ^13^C NMR (100 MHz, CDCl_3_) *δ:* 166.2, 165.2, 155.7, 152.5, 149.9, 149.4, 148.6, 144.8, 137.1, 131.3, 129.0, 124.7, 121.6, 120.0, 116.1, 115.7, 112.6, 112.4, 112.2, 111.1, 92.2, 69.9 (CH_2_), 56.3 (OCH_3_), 54.5 (OCH_3_). *Anal*. Calcd. for C_26_H_21_N_3_O_5_ (455.46): C, 68.56; H, 4.65; N, 9.23%. Found: C, 68.27; H, 4.42; N, 9.51%.

##### 2-(4-(3-Cyano-1-methyl-2-oxo-6-phenyl-1,2-dihydropyridin-4-yl)-2-methoxyphenoxy)-*N*-phenylacetamide (9a):

Pale yellow powder, yield: 0.15 g (30%), mp. 180–182 °C; FT-IR (ATR) *v*_*max*_: 3426 (NH), 3060 (CH_arom._), 2922, 2852 (CH_aliph._), 2220 (CN), 1683 (C = O_amide_) cm^−1^; ^1^H NMR (400 MHz, DMSO-*d*_*6*_) *δ:* 10.29 (s, 1H, NH), 7.65–7.57 (m, 7H, CH_arom._), 7.36–7.30 (m, 4H, CH_arom._), 7.13–7.06 (m, 2H, CH_arom._), 6.51 (s, 1H, CH_arom._), 4.83 (s, 2H, CH_2_), 3.89 (s, 3H, OCH_3_), 3.34 (s, 3H, NCH_3_). ^13^C NMR (100 MHz, DMSO-*d*_*6*_) *δ*: 166.7, 161.6, 157.6, 154.7, 149.9, 149.3, 138.7, 134.5, 130.6, 129.2, 129.0, 128.9, 124.2, 121.8, 120.3, 119.9, 117.3, 114.0, 112.8, 109.0, 98.2, 68.3 (CH_2_), 56.3 (OCH_3_), 35.1 (NCH_3_). *Anal*. Calcd. for C_28_H_23_N_3_O_4_ (465.49): C, 72.24; H, 4.98; N, 9.03%. Found: C, 72.40; H, 4.76; N, 9.21%.

##### 2-(4-(3-Cyano-6-(3-methoxyphenyl)-1-methyl-2-oxo-1,2-dihydropyridin-4-yl)-2-methoxyphenoxy)-*N*-phenylacetamide (9b):

White powder, yield: 0.21 g (40%), mp. 188–190°C; FT-IR (ATR) *v*_*max*_:3392 (NH), 3072 (CH_arom._), 2960, 2940, 2839 (CH_aliph._), 2219 (CN), 1684 (C = O_amide_) cm^−1^; ^1^H NMR (400 MHz, DMSO-*d*_*6*_) *δ:* 10.14 (s, 1H, NH), 7.84–7.79 (m, 1H, CH_arom._), 7.64 (s, 2H, CH_arom._), 7.47–7.45 (m, 1H, CH_arom._), 7.36–7.33 (m, 4H, CH_arom._), 7.18–7.12 (m, 4H, CH_arom_), 6.53 (s, 1H, CH_arom._), 4.82 (s, 2H, CH_2_), 3.89 (s, 3H, OCH_3_), 3.82 (s, 3H, OCH_3_), 3.35 (s, 3H, NCH_3_); ^13^C NMR (100 MHz, DMSO-*d*_*6*_) *δ*: 166.6, 161.5, 159.7, 157.6, 154.4, 149.9, 149.3, 138.8, 135.8, 130.4, 129.2, 128.9, 124.1, 121.8, 120.9, 119.8, 117.4, 116.2, 114.4, 113.9, 112.7, 108.8, 98.1, 68.3 (CH_2_), 56.2 (OCH_3_), 55.8 (OCH_3_), 35.1 (NCH_3_). Dept-135 NMR (100 MHz, DMSO-*d*_*6*_) *δ:* 130.4, 129.2, 124.1, 121.8, 120.9, 119.8, 116.2, 114.4, 113.8, 112.7, 108.8, 68.6 (CH_2_, reversed direction), 56.2 (OCH_3_), 55.8 (OCH_3_), 35.1 (NCH_3_)*. Anal*. Calcd. for C_29_H_25_N_3_O_5_ (495.52): C, 70.29; H, 5.09; N, 8.48%. Found: C, 70.45; H, 5.23; N, 8.32%.

##### 2-(4-(3-Cyano-6-(4-methoxyphenyl)-1-methyl-2-oxo-1,2-dihydro pyridin-4-yl)-2-methoxyphenoxy)-*N*-phenylacetamide (9c):

White powder, yield: 0.21 g (40%), mp. 168–170 °C; FT-IR (ATR) *v*_*max*_: 3394 (NH), 3075 (CH_arom._), 2963, 2940, 2836 (CH_aliph._), 2219 (CN), 1684 (C = O_amide_) cm^−1^; ^1^H NMR (400 MHz, DMSO-*d*_*6*_) *δ*: 10.33 (s, 1H, NH), 7.65 (d, 2H,* J* = 5.3 Hz, CH_arom._), 7.57 (d, 2H,* J* = 7.6 Hz, CH_arom._), 7.35–7.33 (m, 4H, CH_arom._), 7.10 (s, 4H, CH_arom._), 6.48 (s, 1H, CH_arom._), 4.84 (s, 2H, CH_2_), 3.89 (s, 3H, OCH_3_), 3.85 (s, 3H, OCH_3_). ^13^C NMR (100 MHz, DMSO-*d*_*6*_) *δ*: 166.7, 161.9, 161.0, 157.5, 154.8, 149.7, 149.2, 138.6, 130.6, 129.6, 129.3, 129.0, 126.7, 124.3, 121.7, 119.9, 114.6, 113.8, 112.6, 109.1, 97.5, 68.1 (CH_2_), 56.2 (OCH_3_), 55.8 (OCH_3_), 35.3 (NCH_3_). *Anal*. Calcd. for C_29_H_25_N_3_O_5_ (495.52): C, 70.29; H, 5.09; N, 8.48%. Found: C, 70.14; H, 5.26; N, 8.36%.

##### 2-(4-(6-(4-Chlorophenyl)-3-cyano-2-oxo-1,2-dihydropyridin-4-yl)-2-methoxyphenoxy)-*N*-phenylacetamide (9d):

White powder, yield: 0.13 g (25%), mp. 203–205 °C; FT-IR (ATR) *v*_*max*_: 3335 (NH), 3053 (CH_arom._), 2963, 2933, 2848 (CH_aliph._), 2214 (CN), 1683 (C = O_amide_) cm^−1^; ^1^H NMR (400 MHz, DMSO-*d*_*6*_) *δ*: 10.08 (s, 1H, NH), 7.64–7.62 (m, 6H, CH_arom._), 7.36–7.31 (m, 4H, CH_arom._), 7.13–7.07 (m, 2H, CH_arom._), 6.54 (s, 1H, CH_arom._), 4.81 (s, 2H, CH_2_), 3.89 (s, 3H, OCH_3_), 3.34 (s, 3H, NCH_3_). ^13^C NMR (100 MHz, DMSO-*d*_*6*_) *δ:* 166.6, 161.5, 157.5, 153.4, 150.0, 149.4, 138.8, 135.5, 133.4, 130.9, 129.2, 129.2, 128.9, 124.1, 121.8, 119.9, 117.2, 114.1, 112.8, 109.0, 98.5, 68.5 (CH_2_), 56.3 (OCH_3_), 35.0 (NCH_3_). *Anal*. Calcd. for C_28_H_22_ClN_3_O_4_ (499.94): C, 67.27; H, 4.44; N, 8.40%. Found: C, 67.45; H, 4.27; N, 8.58%.

##### 2-(4-(6-(4-Bromophenyl)-3-cyano-1-methyl-2-oxo-1,2-dihydropyridin-4-yl)-2-methoxyphenoxy)-*N*-phenylacetamide (9e):

White powder, yield: 0.10 g (20%), mp. 210–212 °C; FT-IR (ATR) *v*_*max*_: 3426 (NH), 3050 (CH_arom._), 2956, 2924, 2852 (CH_aliph._), 2219 (CN), 1642 (C = O_amide_) cm^−1^; ^1^H NMR (400 MHz, DMSO-*d*_*6*_) *δ*: 10.31 (s, 1H, NH), 8.23(d, 1H,* J* = 8.3 Hz, CH_arom._), 7.77 (d, 2H,* J* = 8.0 Hz, CH_arom._), 7.64 (d, 2H,* J* = 7.7 Hz, CH_arom._), 7.59 (d, 2H,* J* = 8.1 Hz, CH_arom._), 7.36–7.31 (m, 4H, CH_arom._), 7.13–7.11 (m, 1H, CH_arom._), 6.61–6.54 (m, 1H, CH_arom._), 4.83 (s, 2H, CH_2_), 4.15 (s, 3H, OCH_3_), 3.24 (s, 3H, NCH_3_). *Anal*. Calcd. for C_28_H_22_BrN_3_O_4_ (544.39): C, 61.77; H, 4.07; N, 7.72%. Found: C, 61.56; H, 4.24; N, 7.51%.

##### 2-(4-(3-Cyano-1-methyl-6-(naphthalen-2-yl)-2-oxo-1,2-dihydropyridin-4-yl)-2-methoxyphenoxy)-*N*-phenylacetamide (9f):

Pale yellow powder, yield: 0.15 g (30%), mp. 193–195 °C; FT-IR (ATR) *v*_*max*_: 3391 (NH), 3054 (CH_arom._), 2940, 2919, 2854 (CH_aliph._), 2217 (CN), 1691 (C = O_amide_) cm^−1^; ^1^H NMR (400 MHz, DMSO-*d*_*6*_) *δ:* 10.08 (s,1H, NH), 8.20 (s, 1H, CH_arom._), 8.10 (d, 1H, *J* = 8.5 Hz, CH_arom._), 8.03 (d, 2H,* J* = 7.4 Hz, CH_arom._), 7.73–7.62 (m, 5H, CH_arom._), 7.39–7.31 (m, 4H, CH_arom._), 7.14–7.07 (m, 2H, CH_arom._), 6.66 (s, 1H, CH_arom._), 4.81 (s, 2H, CH_2_), 3.89 (s, 3H, OCH_3_), 3.41 (s, 3H, NCH_3_); ^13^C NMR (100 MHz, DMSO-*d*_*6*_) *δ:* 166.6, 161.6, 157.6, 154.6, 150.0, 149.4, 138.8, 133.6, 132.8, 132.0, 129.2, 129.1, 128.9, 128.9, 128.7, 128.2, 128.1, 127.5, 125.8, 124.1, 121.8, 119.9, 117.4, 114.2, 112.9, 109.3, 98.3, 68.5 (CH_2_), 56.3 (OCH_3_), 35.3 (NCH_3_). *Anal*. Calcd. for C_32_H_25_N_3_O_4_ (515.55): C, 74.55; H, 4.89; N, 8.15%. Found: C, 74.78; H, 4.71; N, 8.29%.

##### 2-(4-(3-Cyano-6-(furan-2-yl)-1-methyl-2-oxo-1,2-dihydropyridin-4-yl)-2-methoxyphenoxy)-*N*-phenylacetamide (9 g):

Pale yellow powder, yield: 0.155 g (30%), mp. 170–172°C; FT-IR (ATR) *v*_*max*_: 3373 (NH), 3105 (CH_arom._), 2955, 2921, 2851 (CH_aliph._)_._, 2215(CN), 1683 (C = O_amide_) cm^−1^; ^1^H NMR (400 MHz, CDCl_3_) *δ:* 8.66 (s, 1H, NH), 7.59 (s, 1H, CH_arom._), 7.53 (d, 2H, *J* = 7.9 Hz, CH_arom._), 7.30–7.27 (m, 3H, CH_arom._), 7.16–7.14 (m, 1H, CH_arom._), 7.07 (t, 1H, *J* = 7.3 Hz, CH_arom._), 7.00 (d, 1H, *J* = 8.3 Hz, CH_arom._), 6.85 (d, 1H,* J* = 3.2 Hz, CH_arom._), 6.59 (s, 1H, CH_arom._), 6.55 (s, 1H, CH_arom._), 4.62 (s, 2H, CH_2_), 3.95 (s, 3H, OCH_3_), 3.68 (s, 3H, NCH_3_); ^13^C NMR (100 MHz, CDCl_3_) *δ:* 166.0, 161.5, 157.0, 149.8, 149.7, 149.0, 145.4, 142.7, 138.1, 130.6, 129.1, 124.7, 121.4, 120.0, 115.8, 115.2, 112.3, 112.2, 112.1, 107.4, 93.9, 69.8 (CH_2_), 56.3 (OCH_3_), 34.5 (NCH_3_). *Anal*. Calcd. for C_26_H_21_N_3_O_5_ (455.46): C, 68.56; H, 4.65; N, 9.23%. Found: C, 68.37; H, 4.48; N, 9.45%.

##### 5-cyano-6-methoxy-4-(3-methoxy-4-(2-oxo-2-(phenylamino)ethoxy) phenyl)-1'-methyl-2,3'-bipyridin-1'-iumiodide (10):

Yellow powder, yield: 0.46 g (90%), mp. 284–286 °C; FT-IR (ATR) *v*_*max*_: 3178 (NH), 3061 (CH_arom._), 2952, 2830 (CH_aliph._), 2220 (CN), 1689 (C = O_amide_) cm^−1^; ^1^H NMR (400 MHz, DMSO-*d*_*6*_) *δ:* 10.21 (s,1H, NH), 9.81 (s, 1H, CH_arom._), 9.37 (d, 1H, *J* = 8.1 Hz, CH_arom._), 9.11 (d, 1H, *J* = 5.8 Hz, CH_arom._), 8.31 (t, 1H, *J* = 7.0 Hz, CH_arom._), 8.15 (s, 1H, CH_arom._), 7.64 (d, 2H, *J* = 7.8 Hz, CH_arom._), 7.48 (s, 1H, CH_arom._), 7.40–7.32 (m, 3H, CH_arom._), 7.19–7.08 (m, 2H, CH_arom._), 4.86 (s, 2H, CH_2_), 4.48 (s, 3H, N^+^CH_3_), 4.23 (s, 3H, OCH_3_), 3.92 (s, 3H, OCH_3_). ^13^C NMR (100 MHz, DMSO-*d*_*6*_) *δ:* 166.6, 165.1, 157.2, 150.9, 150.0, 149.6, 146.6, 145.1, 143.1, 138.8, 136.5, 129.1, 128.8, 128.2, 124.1, 122.2, 119.9, 115.8, 115.5, 114.5, 113.5, 95.2, 68.5 (CH_2_), 56.6 (OCH_3_), 55.6 (OCH_3_), 48.9 (N^+^CH_3_)*. Anal*. Calcd. for C_28_H_25_IN_4_O_4_ (608.42): C, 55.27; H, 4.14; N, 9.22%. Found: C, 55.13; H, 4.25; N, 9.08%.

### Biology

#### Anticancer

##### In vitro anti-proliferative assays.

All cell lines were maintained in RPMI1640 medium containing 10% FBS at 37 °C in 5% CO_2_ humidified incubator. Cell proliferation assay was determined by the MTT (3-[4,5-dimethyl-2-thiazolyl]-2,5- diphenyl-2H-tetrazolium bromide) method [39]. Briefly, cells were plated in triplicate wells (3–5 × 10^4^ cells/well) of 96-well flat-bottomed plates and incubated overnight prior to drug exposure and then treated with different concentrations of the tested compounds for 48 h. After that, 20 μL of MTT reagent at a final concentration of 0.5 mg/mL was added to each well. Cells were then incubated for 2 h with MTT, after which 100 μL of DMSO solution was added to dissolve the formazan salt resulting from the reduction of MTT and the absorbance was read at 570 nm using an automatic plate reader [39]. The IC_50_ values were calculated according to inhibition ratios from three independent experiments.

##### PIM-1 kinase inhibitory activity.

The ability of tested compounds for inhibition of PIM-1 kinase enzyme were measured using Promega PIM-1 kinase assay kit (cat # V4032) [40], ADP-Glo™ Reagent and Tecan –spark reader, four concentration (10, 1, 0.1 and 0.01 µM) of the evaluated compounds were prepared using DEMSO as solvent according to prescribed protocol of the manufacturer (Promega corporation, 2800 woods Hollow Road, Madison, WI 537711–5399 USA) [41]. From resulting data IC_50_ for each compound were calculated using GraphPad Prism software.

##### Cell cycle analysis.

Cell cycle analysis MCF-7 cell was seeded into six-well plates at a density of 2 × 10^5^ cell per well and incubated for 24 h. The cell was cultured in RPMI 1640 supplemented with fetal bovine serum (FBS, 10%) and incubated at 37 °C and 5% CO_2_. The medium was removed and replaced with medium (final DMSO concentration, 1% v/v) containing compound **7h** (0.283 µM). After incubation for 24 h and 48 h, the cell layer was trypsinized and washed with cold phosphate buffered saline (PBS) and fixed with 70% ethanol. The fixed cells were rinsed with PBS and then stained with the DNA fluorochrome PI in a solution containing Triton X-100 as well as RNase, keep 15 min at 37 °C according to the instruction manual. Then the samples were analyzed with a FACS Caliber flow cytometer (Becton Dickinson & Co., Franklin Lakes, NJ). The number of cells analyzed for each sample was 10,000 [39].

##### Apoptosis assay.

The MCF-7 was treated with compound **7h** for 24 h. After treatment, the cells were suspended in 0.5 mL of PBS, collected by centrifugation, and fixed in ice-cold 70% (v/ v) ethanol, centrifuged the ethanol-suspended cells for 5 min, suspended in 5 mL PBS and centrifuged for 5 min, re-suspended with 1 mL PI staining solution (0.1 mg/ml RNase) + PE Annexin V (component no. 51-65875X) and kept in dark at 37 °C for 10 min, finally analyzed by flow cytometry using FACS caliber (Becton Dickinson). The cell cycle distributions were calculated using Phoenix Flow Systems and Verity Software House [39].

##### Molecular docking methodology.

AutoDock Vina v.1.2.0 was used for carrying out the molecular docking [42–44]. For more details see supporting information.

## Data Availability

No datasets were generated or analysed during the current study.
